# Tuning the Surface Activity and Micellization of *closo*-Dodecaborate-Based Dianionic Surfactants via Linker and Counterion Selection

**DOI:** 10.1021/acs.langmuir.5c04598

**Published:** 2025-11-27

**Authors:** Belhssen Hleli, Peter Ogrin, Zdeněk Tošner, Žiga Medoš, Tomáš Křížek, Bojan Šarac, Tomaž Urbič, Marija Bešter-Rogač, Pavel Matějíček

**Affiliations:** † Department of Physical and Macromolecular Chemistry, Faculty of Science, 37740 Charles University, Hlavova 2030/8, 128 40 Prague 2, Czech Republic; ‡ Faculty of Chemistry and Chemical Technology, 37663 University of Ljubljana, Večna pot 113, SI-1000 Ljubljana, Slovenia; § NMR Laboratory, Faculty of Science, 37740 Charles University, Hlavova 2030/8, 128 40 Prague 2, Czech Republic; ∥ Department of Analytical Chemistry, Faculty of Science, 37740 Charles University, Hlavova 2030/8, 128 40 Prague 2, Czech Republic

## Abstract

To understand the impact of alkaline counterions on the self-assembly of atypical dianionic surfactants, we synthesized a series of novel amphiphilic compounds featuring a bulky dianionic head based on *closo*-dodecaborate and a hydrophobic alkoxy tail connected via short linkers of different complexation ability toward alkaline cations. The synthetic strategy relied on the opening of cyclic oxonium derivatives by activated alcohol, enabling the attachment of hydrophobic chains to the boron cluster framework. The synthesis of novel surfactants with high cationic purity free from contaminating ions or additional inorganic salts is described. Self-assembly of the dodecaborate-based surfactants at the air–water interface and in aqueous solutions was investigated using tensiometry, various NMR spectroscopy techniques, dynamic light scattering, and all-atom MD simulations. Interestingly, despite their dianionic heads, the surfactants form compact monolayers in cases in which efficient binding of counterions to surfactant dianions occurs. MD simulations suggested relatively small surfactant micelles above the critical micelle concentration, which was confirmed by light scattering analysis. There is evidence that their aggregation number and morphology are strongly dependent on counterion immobilization. The mobility of counterions was observed directly by ^7^Li, ^23^Na, ^39^K, and ^133^Cs NMR spectroscopy, which allowed us to understand the binding abilities of linkers and dianionic heads toward counterions.

## Graphical abstract

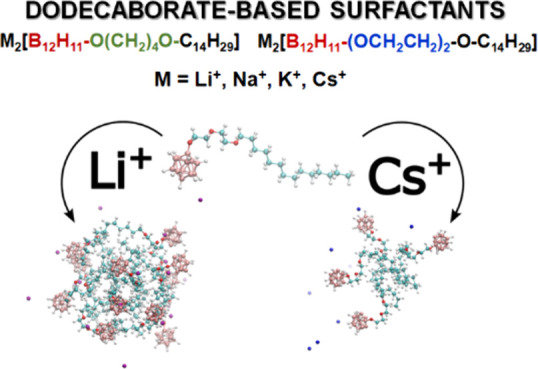

## Introduction

Amphiphilic molecules or ions are surface-active species characterized by distinct polar (hydrophilic) and nonpolar (hydrophobic) regions, which drive the formation of micelles in dilute aqueous solutions. Although the basic principles of the solution behavior of surfactants have been thoroughly studied and reported,^1–5^ newly synthesized and investigated borderline cases can help us to better understand the process and driving forces of surfactant micellization, which in turn could broaden the application potential of amphiphilic species.^6–9^

Surfactants are already of eminent importance in not only industrial and advanced applications but also everyday life.^5^ Their amphiphilic nature leads to preferential accumulation at the water–air and water–oil interfaces, resulting in the formation of stable foams and emulsions. Apparently, the formation of micelles has a dampening effect on detergent properties of surfactants since the molecules involved in micellization no longer contribute to surface activity.^3^ However, the surfactant micelles can serve as hydrophobic domains in aqueous media suitable for delivery of water insoluble compounds^5^ or as templates in advanced nanotechnologies.^10^

A closed association model describing the self-assembly of classical surfactants predicts a spontaneous step-like formation of multimolecular micellar aggregates at the critical micelle concentration (CMC).^1–4^ Similarly, when mass action modeling is applied, at sufficiently large aggregation numbers (>50), one can describe the micellization by one equilibrium constant, allowing estimation of the free energy of micellization directly from the CMC value.

Systematic thermodynamic studies based on isothermal titration calorimetry (ITC), however, revealed that this simple one-step model often fails to accurately describe the real micellization even of simple surfactants, when the alkyl chain lengths are shorter (≤12 carbon atoms).^11–14^ This is also true for the aggregation behavior of nonclassical amphiphiles, anionic metallacarboranes (COSAN and FESAN), where a two-step model was required for fitting the ITC data.^15,16^ The metallacarborane aggregation resembles in part the surfactant micellization but with several important differences, including a very low aggregation number and an enthalpy-driven aggregation process strongly influenced by counterions.^8^

As indicated above, even though a hydrophobic effect has a pivotal role in the formation of surfactant assemblies in aqueous solution in bulk and at interfaces, (counter)­ions, although often neglected, play an important role in the self-assembly of surfactants and other amphiphiles, as well. According to the manner of action, we can sort the impact of ions into several groups: (i) counterion binding to the micelles/aggregates of ionic surfactants or anionic boron cluster compounds to partly compensate for the charge of nanostructures,^9,11,15–18^ (ii) ion-specific (Hofmeister) effects, in regard to monolayers and nanoparticles, acting beyond simple electrostatics,^19–23^ (iii) superchaotropic effect established recently as a separate category (an extreme case of Hofmeister effects),^7,24^ and (iv) interaction of ions (such as organic ones) with surfactant micelles via diverse weak interactions.^11,17,18^

In our previous study, we reported the synthesis of novel, atypical surfactants with a conventional amphiphilic structure but with the dianionic *closo*-dodecaborate head.^9^ This system challenged the concept of step-like micellization. A three-step thermodynamic model (three equilibria for micelle formation) was used for treatment of the ITC data for the first time, and the concept of step-like micellization with a singular CMC was found to be inappropriate. It was evident that the micellization is complex, and the micellar structure is a result of an intricate balance between hydrophobic effect-driven micellization and extensive counterion binding. However, the model failed to fit the experimental data at all temperatures simultaneously due to the complex premicellar aggregation approximated at the time by empirical corrections of the applied model. Premicellar aggregates challenging the simplified concept of surfactant micellization were experimentally detected even for conventional surfactants.^25^ A separate publication is in preparation to provide further detailed ITC experimental data and analyses of the selected samples presented in this work.

Anionic boron cluster compounds, including metallacarboranes and stable *closo*-borates, are purely inorganic polyhedral species of nanometer size with a peculiar three-dimensional aromatic structure and complex solution behavior.^26–29^ Besides their aggregation tendencies and surface activity,^7,8,30–34^ they exhibit superchaotropic behavior^35–39^ and are involved in dihydrogen bonding,^8,17^ enabling the formation of novel boron-rich nanostructures.^29^ Boron cluster compounds are intensively studied in medicinal research (BNCT cancer therapy and antiviral and antimicrobial properties)^28,40–44^ and advanced material research (ion-conducting materials, photoluminescence and optoelectronics, and thermoresistant materials).^29^

To further understand all of the factors influencing the self-assembly of anionic boron cluster-based surfactants, we conducted a follow-up study,^9^ in which we synthesized a series of B12-linker-tail structures (Scheme 1) with different counterions (Li, Na, K, and their blends with a small fraction of Cs) and various linkers between charged heads and hydrophobic tails. Previously,^9^ only sodium salts were studied, and the linker was based on the opening of the dioxanate ring attached to *closo*-dodecaborate clusters, which resulted in a short linker containing three oxygen atoms. In the current study, we also tested a linker containing two oxygen atoms based on the opening of the oxonium THF ring. We focused on the synthesis of surfactants of excellent cationic purity, and in this regard, we had to change the synthetic protocol used previously.^9^ Then, we investigated the surface activity of the novel surfactants and their ability to form monolayers at the air–water interface. Micellization behavior, including the mobility of dianions and counterions as well as micelle size and morphology, was studied by ^1^H, ^7^Li, ^23^Na, ^39^K, and ^133^Cs NMR spectroscopy and all-atom MD simulations in explicit water. The presented study should contribute to a deeper understanding of the surfactant micellization process, which can be tuned by a selection of counterions.^11,17,18^

**Scheme 1:**
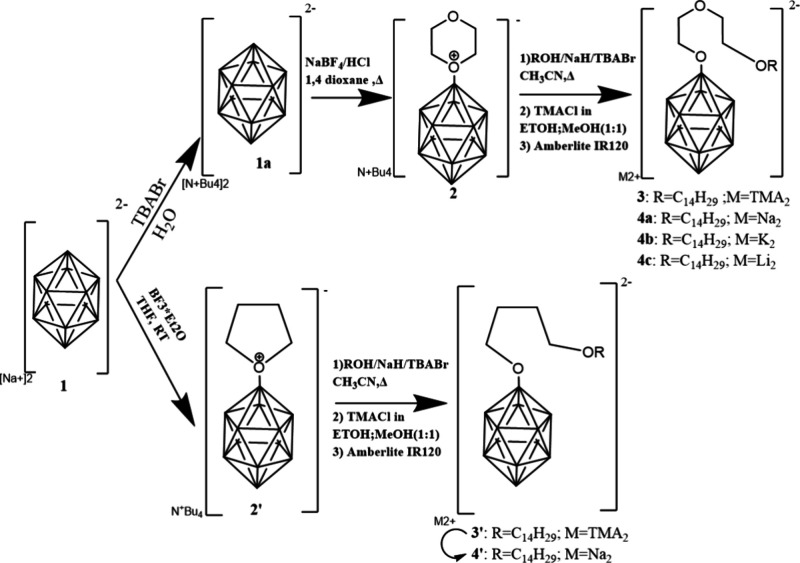
Synthetic Route to X_2_[B_12_H_11_-linker-C_14_H_29_] (X = Li, Na, and K) Starting from Na_2_[B_12_H_12_] (**1**) via [Bu_4_N]­[B_12_H_11_-dioxanate] (**2**) to M­[B_12_H_11_-(OCH_2_CH_2_)_2_-O-R] (R = C14, M = TMA_2_ (**3**), M = Na_2_ (**4a**), M = K_2_ (**4b**), M = Li_2_ (**4c**)) and via [Bu_4_N]­[B_12_H_11_-THF] (**2′**) to M­[B_12_H_11_-O­(CH_2_)_4_-O-R] (R = C14, M = TMA_2_ (**3′**), M = Na_2_ (**4′**))

## Experimental Section

### Materials

Disodium *closo*-dodecaborate, anhydrous, Na_2_[B_12_H_12_], was purchased from KatChem Ltd. and used without any purification. For the synthesis of oxonium conjugates, for the THF linker (OC4), sodium salt was used as purchased (**1**). However, for the dioxane linker (EO2), disodium salt was converted into [Bu_4_N]_2_[B_12_H_12_] salt (**1a**) by precipitation with 2.1 equiv of [Bu_4_N]Br in aqueous solutions.

Hydrochloric acid in 1,4-dioxane, tetrabutylammonium bromide, sodium tetrafluoroborate, sodium hydroxide, potassium hydroxide, lithium hydroxide, sodium hydride, tetradecanol, boron trifluoride etherate, and Amberlite (IR120 Na^+^ form) were received from Aldrich and used as purchased.

### Synthesis

#### [Bu_4_N]­[B_12_H_11_-dioxanate] (**2**)

**2** was synthesized and purified as described in our previous work.^9^ Briefly, 1.0 mL of a 4 M HCl solution in 1,4-dioxane was added to a round flask under nitrogen along with a suspension of 1.25 g (2.0 mmol) of [Bu_4_N]_2_[B_12_H_12_] (**1a**) and 1.10 g (10.0 mmol) of Na­[BF_4_] in 70 mL of 1,4-dioxane to afford 0.8 g of a white product (yield 85.1%): ^1^H NMR (acetone-*d*_6_) δ 4.52 (4H, m), 3.87 (4H, m), 3.1 (8H, m, [Bu_4_N]^+^), 1.61 (8H, m, [Bu_4_N]^+^), 1.37 (8H, m, [Bu_4_N]^+^), 0.99 (12H, t, [Bu_4_N]^+^); ^11^B­{^1^H} NMR (acetone-*d*_6_) δ 8.83 (1B), −16.9 (5B), −17.57 (5B), −19.76 (1B).

#### [TMA]_2_[B_12_H_11_-(OCH_2_CH_2_)_2_-O-C_14_H_29_] (**3**)

To a solution of 10 mmol of tetradecanol in 25 mL of dry CH_3_CN was added 0.51 g (10 mmol) of NaH, and the mixture was stirred for 5 min until the evolution of H_2_ had stopped. One gram of **2** (2 mmol) and 2 mmol of tetrabutylammonium bromide were added, and the mixture was refluxed under nitrogen for 10 h. After the reaction had reached completion, the solid was filtered off, and the solvent was evaporated to give **3** as a yellowish oil. This crude product was then dissolved in a 1:1 MeOH/EtOH mixture followed by the addition of 2.1 equiv of [TMA]Cl to obtain a white precipitant, which was filtered, purified three times by reprecipitation from ethanol, and dried overnight to afford **3** as a white product (yield 70%): ^1^H NMR (CD_3_CN) δ 3.51 (8H, m), 3.43 (2H, t), 3.12 (3H, s), 1.54 (2H, m), 1.3 (22H, m), 0.9 (3H, t); ^11^B­{^1^H} NMR (CD_3_CN) δ 6.64­(1B), −16.24 (5B), −18.15­(5B), −23.37 (1B).

#### Preparation of M_2_[B_12_H_11_-(OCH_2_CH_2_)_2_-O-C_14_H_29_] (M = Na^+^, K^+^, and Li^+^) (**4a–c**, respectively)

Na_2_[B_12_H_11_-(OCH_2_CH_2_)_2_-O-C_14_H_29_] was prepared as follows. The [TMA]^+^ form was dissolved in a 1:1 acetonitrile/water mixture and then passed through a column filled with Amberlite Na^+^. The eluent was concentrated under reduced pressure to remove the acetonitrile, and the remaining aqueous solution was lyophilized to afford **4a** as a white product (yield 60%): ^1^H NMR (D_2_O) δ 3.58 (8H, m), 3.46 (2H, t), 1.52 (2H, m), 1.23 (22H, m), 0.81 (3H, t); ^11^B­{^1^H} NMR (D_2_O) δ 6.64 (1B), −16.24 (5B), −18.34 (5B), −23.37 (1B).

M_2_[B_12_H_11_-(OCH_2_CH_2_)_2_-O-C_14_H_29_] (M = K^+^ and Li^+^) was prepared as follows. The Amberlite Na^+^ filling a column was exchanged for the M^+^ form (K^+^ or Li^+^) by passing a MOH solution (KOH or LiOH) through the column, and then it was neutralized by adding a HCl solution until the pH reached 7. The [TMA]^+^ salt of the compound was then processed through this column as described for Na^+^ exchange. K_2_[B_12_H_11_-(OCH_2_CH_2_)_2_-O-C_14_H_29_] (**4b**) was a white powder (yield 60%), and Li_2_[B_12_H_11_-(OCH_2_CH_2_)_2_-O-C_14_H_29_] (**4c**) was a white powder (yield 60%).

**4b**: ^1^H NMR (D_2_O) δ 3.58 (8H, m), 3.45 (2H, t), 1.53 (2H, m), 1.23 (22H, m), 0.83 (3H, t); ^11^B­{^1^H} NMR (D_2_O) δ 6.64 (1B), −16.43 (5B), −18.15 (5B), −23.18 (1B).

**4c**: ^1^H NMR (D_2_O) δ 3.58 (8H, m), 3.46 (2H, t), 1.52 (2H, m), 1.22 (22H, m), 0.81 (3H, t); ^11^B­{^1^H} NMR (D_2_O) δ 6.64 (1B), −16.24 (5B), −18.34 (5B), −23.37 (1B).

#### [Bu_4_N]­[B_12_H_11_-THF] (**2′**)

A 1.48 mL (11.62 mmol) portion of BF_3_·Et_2_O was added to a solution of 2 g (10.52 mmol) of Na_2_B_12_H_12_ in 50 mL of dry THF in a round flask under nitrogen and then stirred at room temperature for 12 h. The solution was filtered and dried under reduced pressure. The residue was dissolved in 100 mL of water and treated with a solution of 6.78 g (21.03 mmol) of tetrabutylammonium bromide in 50 mL of water. The formed precipitate was filtered and dried in air to afford 3.4 g as a white product (yield 70%): ^1^H NMR (acetone-*d*_6_) δ 4.43 (4H, t, -O­(CH_2_CH_2_)_2_), 3.11 (8H, t, Bu_4_N^+^), 2.15 (4H, t, -O­(CH_2_CH_2_)_2_), 1.61 (8H, m, Bu_4_N^+^), 1.38 (8H, m, Bu_4_N^+^), 0.99 (12H, t, Bu_4_N^+^); ^11^B­{^1^H} NMR (acetone-*d*_6_) 6.7 (1B, s), −17.11 (10B, s), −19.94 (1B, s).

#### Preparation of Na_2_[B_12_H_11_-O­(CH_2_)_4_O-C_14_H_29_] (**4′**)

Na_2_[B_12_H_11_-O­(CH_2_)_4_-O-C_14_H_29_] was prepared as follows. The [TMA]^+^ form was dissolved in a 1:1 acetonitrile/water mixture and then passed through a column filled with Amberlite Na^+^. The recovered solution was dried under reduced pressure to remove the acetonitrile, and the remaining aqueous solution was lyophilized to afford **4′** as a white product (yield 60%): ^1^H NMR (D_2_O) δ 3.43 (6H, m), 1.49 (6H, m), 1.23 (22H, m), 0.83 (3H, t); ^11^B­{^1^H} NMR (D_2_O) δ 6.38 (1B), −16.33 (5B), −18.35 (5B), −23.42 (1B).

### Methods

#### NMR Spectroscopy

NMR spectra were measured on a Bruker Avance III spectrometer operating at ^1^H Larmor frequencies of 400 and 600 MHz using acetone-*d*_6_ and deuterium oxide solutions (99.5%; Chemotrade, Leipzig, Germany). ^1^H NMR spectra were referenced to the residual solvent signals (2.04 and 4.80 ppm for acetone and water, respectively). ^1^H DOSY NMR measurements were performed with the double stimulated echo experiment with bipolar pulse field gradients (dstebpgp3s pulse program)^45^ using 32 gradient values linearly spaced between 2% and 98% of the maximal value (50 G/cm), a 300 ms diffusion delay (D20), and a 1.5 ms gradient duration (P30). The calculation of the absolute values of diffusion constants was calibrated using 1% H_2_O in a D_2_O sample with a HDO diffusion constant of 1.9 × 10^–9^ m^2^/s. ^23^Na NMR spectra and *T*_1_^23^Na NMR relaxation times were measured at a resonance frequency of 158.7 MHz using up to 128 scans and 16 delays in the inversion recovery experiment (recycle delay of 1 s). All measurements were conducted at 298.15 K. Data processing and fitting of the diffusion coefficients and relaxation times were performed by using MestReNova or Topspin software. *T*_1_ values were recalculated to afford diffusion constants using the formula given by D’Agostino et al.^46^ The validity of this approach was verified by independent measurements of a 10 mM NaCl solution and by comparing the evaluated diffusion constant of 11.2 × 10^–10^ m^2^/s to the literature value (14 × 10^–10^ m^2^/s for 30 mM NaCl).^47^ ^7^Li NMR spectra were measured at a resonance frequency of 233.2 MHz using a recycle delay of 50 s with 16 scans. In ^7^Li DOSY NMR experiments (dstebpgp3s pulse program), these parameters were used: 16 gradient values linearly spaced between 2% and 98% of the maximal value, 150 ms diffusion delay (D20), and 2 ms gradient duration (P30). Absolute translation diffusion coefficients were not calculated, and only relative values were evaluated by the Stejskal–Tanner equation. ^39^K NMR spectra were measured at a resonance frequency of 28.0 MHz and a recycle delay of 0.1 s with 10 240 scans. *T*_2_ values were estimated from the peak width at half-height. ^133^Cs NMR spectra were measured at a resonance frequency of 78.7 MHz and a recycle delay of 30 s with 128–256 scans. In ^133^Cs DOSY experiments (dstebpgp3s pulse program), these parameters were used: 16 gradient values linearly spaced between 2% and 98% of the maximal value, 300 ms diffusion delay (D20), and 2.5 ms gradient duration (P30).

#### Tensiometry

The surface tensions of B12-tail surfactant aqueous solutions in the concentration range of 0.01–80 mM were measured by means of the pendant drop method using an Attension optical tensiometer (Biolin Scientific AB, Sweden) at 25 °C. Calibration was performed with pure deionized water with a surface tension of 72.0 mN/m. A Hamilton microsyringe with a needle nominal inside diameter of 0.413 mm was used. Dynamic surface tension (DST) data were recorded in triplicate for 20 s at each sample concentration. Each DST data set was averaged, and then the resulting surface tension was obtained as the mean value of three independent measurements. The pre-CMC data were processed by methods assuming the Gibbs adsorption isotherm with the factor *n* = 3. The value of the CMC was estimated by the intersection of the lines before and after the break. The pre-CMC regions were fitted by second-order polynomial functions. The area per molecule (APM) was calculated from the slope of γ versus ln *c* dependence according to the Gibbs approach.

#### Capillary Zone Electrophoresis (CZE)

Electrophoretic experiments were carried out on an Agilent 7100 CE system (Agilent Technologies, Waldbronn, Germany) equipped with a TraceDec contactless conductivity detector (Innovative Sensor Technologies, Austria). An unmodified fused silica capillary (Polymicro Technologies, Phoenix, AZ) (20 μm inside diameter, 375 μm outside diameter, 80.0 cm total, and 65.0 cm effective length) was used. Between individual runs, the capillary was flushed for 5 min with a background electrolyte, i.e., 1 M formic acid. The cassette temperature was set to 25 °C. Samples were injected using a pressure of 5 kPa for 20 s. A voltage of 30 kV (current of approximately 4 μA) was applied during the separation of cations (cesium, potassium, sodium, lithium, tetramethylammonium, tetraethylammonium, and tetrabutylammonium). For the separation of anions (*closo*-dodecaborate, chloride, and tetrafluoroborate), a voltage of −30 kV was applied; all other conditions were identical. For the separation of carbonate and phosphate, an unmodified fused silica capillary (50 μm inside diameter, 375 μm outside diameter, 80.0 cm total, and 71.5 cm effective length) was used. Before each run, the capillary was flushed for 2 min with a background electrolyte, consisting of 20 mM pyridinedicarboxylic acid, adjusted with ammonia to pH 9.0, with the addition of 0.5 mM cetyltrimethylammonium chloride. The cassette temperature was set to 25 °C. Samples were injected using a pressure of 5 kPa for 5 s. A voltage of −25 kV (current of approximately 32 μA) was applied during the separation. UV detection at 211 nm was used.

#### Dynamic Light Scattering (DLS)

The light scattering photometer (ALV, Germany) consisted of a model CGS-3 automatic goniometer, a model 7004 multitau multibit autocorrelator, two high-QE APD pseudo-cross-correlation detectors, and a 100 mW diode-pumped solid-state laser with a wavelength λ of 660 nm. DLS measurements of the surfactant micelles in water were performed at different scattering angles (50–150° with an angular step 10°) and 25 °C. DLS data were evaluated by a treatment of the measured normalized intensity autocorrelation function *g*_2_(*t*) = 1 + β|*g*_1_(*t*)|^2^, where *g*_1_(*t*) is the electric field correlation function, *t* is the lag time, and β is a factor accounting for the deviation from the ideal correlation. Using ALV software, an inverse Laplace transform of *g*_1_(*t*) with the aid of a constrained regularization algorithm (CONTIN) provided the distribution of relaxation times, *τA*(τ). Effective angle- and concentration-dependent hydrodynamic radii, *R*_H_(*q*, *c*), were obtained by the ALV software from the mean values of relaxation times, τ_m_(*q*, *c*), of individual modes by using the Stokes–Einstein equation. For individual surfactant micelles, the mean hydrodynamic radii were calculated by averaging the values obtained for all of the measured angles. All of the solutions were filtered by PVDF 0.1 μm filters prior to the measurements.

#### All-Atom Molecular Dynamics Simulations (MD simulations)

We used the Universal Force Field (UFF).^48^ The OBGMX web service^49^ was used to appoint force field parameters for B12-EO2-C14 and B12-OC4-C14 ions. However, partial charges of atoms were calculated by using QM methods. For water, the SPC/E model was used. Molecular dynamics simulations were performed with the GROMACS 2020.3 simulation package.^50^ All atoms in the simulation box were treated explicitly. Periodic boundary conditions in all directions were used. The integration step size was 2 fs. Bonds involving hydrogen were constrained using the LINC algorithm. Cutoff distances for electrostatic and van der Waals interactions were set to 1.0 nm. The PME method was used for long-range electrostatic interactions. The temperature was regulated using stochastic velocity rescaling, while the coupling constant was 0.1 ps. Stochastic cell rescaling was used for pressure control, with a target pressure of 1.0 bar, a coupling constant of 2.0 ps, and a compressibility of 4.5 × 10^–5^ bar^–1^. The number of B12-linker1/2-C14 ions in the simulation box was always 100. The number of cations was such that the system was net neutral. The number of solvent molecules was chosen in such a way that the target concentration of the surfactant was reached. After particles were inserted into the box, energy minimization was carried out to prevent the formation of high-energy structures. Then *NVT* equilibration was performed for 100 ps, and then, *NPT* equilibration was performed for 500 ps. The sampling part of the simulation followed, which was 40 ns long.

## Results and Discussion

### Synthesis of Alkaline Salts of Surfactants B12-EO2-C14 and B12-OC4-C14

A series of alkaline salts of novel surfactants consisted of *closo*-dodecaborate heads, B12, di­(oxy-ethylene), EO2, or oxy-tetramethylene linkers, OC4, and aliphatic -O-C_14_H_29_ tails, C14, (Scheme 1; full systematic formulas in the Experimental Section and section 1.1 of the Supporting Information), abbreviated hereafter as follows. Li2­[B12-EO2-C14] (**4c**), Na2­[B12-EO2-C14] (**4a**), K2­[B12-EO2-C14] (**4b**), and Na2­[B12-OC4-C14] (**4′**) were prepared by the established technique for functionalization of *closo*-dodecaborate via a ring-opening reaction of a cyclic oxonium–dodecaborate conjugate with an alkoxy group^9,51–54^ (Scheme 1) (NMR characterization and further details in the Experimental Section and Figure S1a–g).

The critical challenge of our study was to prepare samples of excellent cationic purity with practically zero contamination by ions used in the synthetic steps (especially alkylammonium cations and Cs^+^) without any additional inorganic salts (such as NaOH or NaCl after neutralization of excess hydroxide by HCl). To achieve this, we attempted to avoid the synthetic path used in our previous study and elsewhere,^9,53^ which required a conversion to triethylammonium salts via tetrabutylammonium (TBA^+^) and water insoluble cesium salts, where the desired alkaline salts were achieved by a careful titration with solutions of the corresponding alkaline hydroxides leading to a release of volatile triethylamine. This method eventually may lead to products of acceptable purity but with low yields and tedious multistep purification. Furthermore, these samples almost always contained traces of Cs^+^. Therefore, we optimized the ion exchange procedure via a column filled with Amberlite resins in the Na^+^, K^+^, and Li^+^ cycles (details in the Experimental Section). The presence or absence of the corresponding alkaline cations as well as undesired ions (such as Cs^+^, TBA^+^, BF_4_^–^, B_12_H_12_^2–^, and Cl^–^) was determined by CZE. The analysis detected no additional ions on a qualitative level in any of the newly synthesized samples. Nevertheless, to complement the study and to understand better the impact of cations on surfactant micellization, we also investigated Li2/Cs­[B12-EO2-C14] and K2/Cs­[B12-EO2-C14] cationic blends prepared by a previously used procedure^9^ containing a fraction of Cs^+^ determined by CZE to be ca. 8 mol % for both samples. The pure Cs salts of B12-linker-tail surfactants are insoluble in water (solubility of <1%). However, the Cs2­[B12-EO2-C14] system was investigated by means of MD simulations to better understand ion-specific effects standing behind the micellization of *closo*-dodecaborate-based surfactants.

The pure samples are highly hygroscopic, white fluffy solids. The determination of the exact sample concentrations was critical for physicochemical studies. Thermogravimetric analysis (TGA) and inductively coupled plasma mass spectrometry (ICP-MS) did not provide convincing data about the water content of the solid and the boron concentration in solutions, respectively. Eventually, we found weighing the freshly lyophilized solid samples sealed in flasks to be the most reliable route for the preparation of defined sample aqueous solutions.

### Surface Activity of B12-EO2-C14 and B12-OC4-C14

The self-assembly properties at the air–water interface of the newly synthesized series of *closo*-dodecaborate-based surfactants were investigated by the pendant drop technique, and all of the results are shown in Figure 1 (chemical structures of the surfactants shown as insets of Figure 1a), Table 1, and Figure S2. The surface tension (γ) curves (Figure 1a) follow the usual trends for ionic surfactants, and they are indicative of the formation of Gibbs monolayers (decrease in γ in the pre-CMC region) and formation of micelles above the CMC (breaks in γ vs ln *c* dependences). The values of CMC, the area per molecule (APM), and the limiting surface tension (γ_CMC_) for all of the studied samples are listed in Table 1 (details in the Experimental Section). We stress that the absence of distinct minima around the CMC confirms that we almost achieved the samples without any surface-active contaminants.

**Figure 1:**
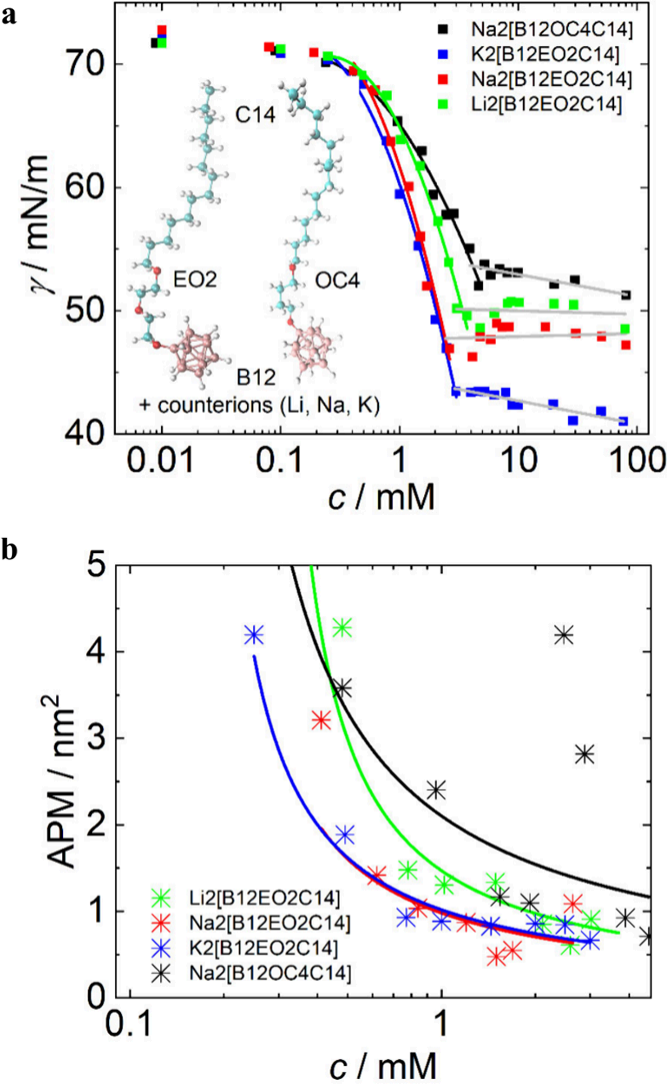
(a) Surface tension (γ) of aqueous solutions of Li2­[B12-EO2-C14] (green), Na2­[B12-EO2-C14] (red), K2­[B12-EO2-C14] (blue), and Na2­[B12-OC4-C14] (black) measured by the pendant drop method at 25 °C. The pre-CMC regions were fitted by second-order polynomial functions (solid lines). Structures of B12-EO2-C14 and B12-OC4-C14 shown as insets. Color coding: salmon for B, red for O, cyan for C, and white for H. (b) Area per molecule (APM) for Li2­[B12-EO2-C14] (green), Na2­[B12-EO2-C14] (red), K2­[B12-EO2-C14] (blue), and Na2­[B12-OC4-C14] (black) calculated from the surface tension data (asterisks) and their second-order polynomial fits (solid curves).

**Table 1:** Values of the Area per Molecule (APM), Critical Micelle Concentration (CMC), and Limiting Surface Tension (at the CMC) (γ_CMC_) of Li2­[B12-EO2-C14], Na2­[B12-EO2-C14], K2­[B12-EO2-C14], and Na2­[B12-OC4-C14] Samples in Water at 25 °C Obtained by Tensiometry (pendant drop technique) (evaluation in Figure S2a–d)

sample	APM (nm^2^)	CMC (mM)	γ_CMC_ (mN/m)
Li2[B12-EO2-C14]	0.8 ± 0.1	3.4 ± 0.6	50.1 ± 1.4
Na2[B12-EO2-C14]	0.6 ± 0.2	2.4 ± 0.9	47.7 ± 1.2
K2[B12-EO2-C14]	0.6 ± 0.1	2.9 ± 0.4	43.7 ± 0.6
Na2[B12-OC4-C14]	1.2 ± 0.6	4.1 ± 1.3	53.6 ± 1.3

The assumed structure of B12-linker-tail monolayers was described previously.^9^ Now, we will focus on the impact of cations and linkers on the surface activity of the samples. Despite the presence of dianionic heads (structures of the surfactants in Figure 1a), the APM of the samples with EO2 linkers (Figure 1b) is relatively low and comparable to that of sodium tetradecyl sulfate: APM = 0.5 nm^2^, CMC = 2.1 mM, and γ_CMC_ = 40 mN/m.^6,9^ It indicates efficient shielding of the −2 charge by counterions, allowing the formation of compact Gibbs monolayers.

It is interesting to note that for alkaline salts of X2­[B12-EO2-C14], the Li sample shows the highest value of APM in comparison to the Na and K samples. This dependence is somehow in line with data published for 1:1 alkaline salts of various anionic surfactants by Lunkenheimer et al. and others,^55–59^ where the least hydrated counterions such as Cs^+^ promote the formation of monolayers with the smallest APM values and the lowest γ_CMC_ values. This indicates the closest packing of the surfactant anions, making such samples the most surface active within the series. In contrast, surfactants with highly hydrated Li^+^ resulted in the least surface-active systems in the series with the highest APM values, because a repulsion between the charged heads is shielded by counterions less efficiently in this case. In this approach, the packing of anionic surfactants is dictated by the hydration radius of counterions, where hydrated Li^+^ is larger than hydrated Cs^+^ or K^+^.

Interestingly, the Na2­[B12-OC4-C14] sample is the least surface-active compound among the synthesized samples, with an APM value almost twice as high as that of its EO2-linker analogue. This indicates that the simple model of packing of anionic surfactants directed by a hydration radius of counterions mentioned above^57–59^ might not be universal. We can speculate that the EO2 linker participates in the binding of alkaline cations within the monolayer, whereas the OC4 linker is less efficient in this regard. To confirm this statement, further investigations should be performed with a broad alkaline salt series of the OC4-containing surfactants, which is, however, beyond the scope of the current study. Another factor could be that the OC4 linker is more hydrophobic and thus may occupy a larger area at the interface in comparison to the conformation of EOn linkers, which adopt more stretched conformations toward the aqueous phase.^9,60,61^

### Mobility of B12-EO2-C14 and B12-OC4-C14 Dianions and Their Counterions in Water

As shown above, all of the studied surfactants exhibited a break in the surface tension versus concentration dependences, indicating the formation of micelles above the CMC (Figure 1a and Figure S2). The visualization and basic description of the Na2­[B12-EO2-C12/14] micelles as well as the micellization process with all of the peculiarities of these types of surfactants were reported previously.^9^ Now, we will focus on the differences within the series of alkaline salts as well as the impact of the number of oxygens in the linker.

We have been guided by our experience that NMR spectroscopy techniques, in particular DOSY NMR, are indispensable tools for the investigation of the self-assembly properties in solutions of small molecules and ions, including anionic boron cluster compounds and surfactants.^9,16,31,32^ We leveraged the great versatility of NMR spectroscopy and used not only basic ^1^H and ^11^B spectroscopies but also ^7^Li, ^23^Na, ^39^K, and ^133^Cs NMR for tracking the counterions in a concentration series of the studied surfactants in the range of ca. 1–25 mM. These techniques not only provide insight into the chemical surroundings of the inspected nuclei (chemical shifts δ) but also enable the detection of changes in the dynamics of counterions during the micellization.

The results of the NMR study are shown in Figures 2 and 3 and Figures S3 and S4. Even simple 1D NMR spectra are sensitive to aggregation in solution^31^ (changes in the δ and width of spectral lines), as shown in Figure 3 and Figure S4, in ^1^H, ^7^Li, and ^133^Cs NMR spectra, albeit such changes are only mild within the experimental error for nuclei with few electrons. In contrast, we found ^133^Cs NMR spectroscopy was exceptionally sensitive as ^133^Cs is electron-rich, which results in large changes in chemical shifts (around 20 ppm). Nevertheless, without additional information, the 1D NMR data are difficult to interpret in all of the cases.

**Figure 2:**
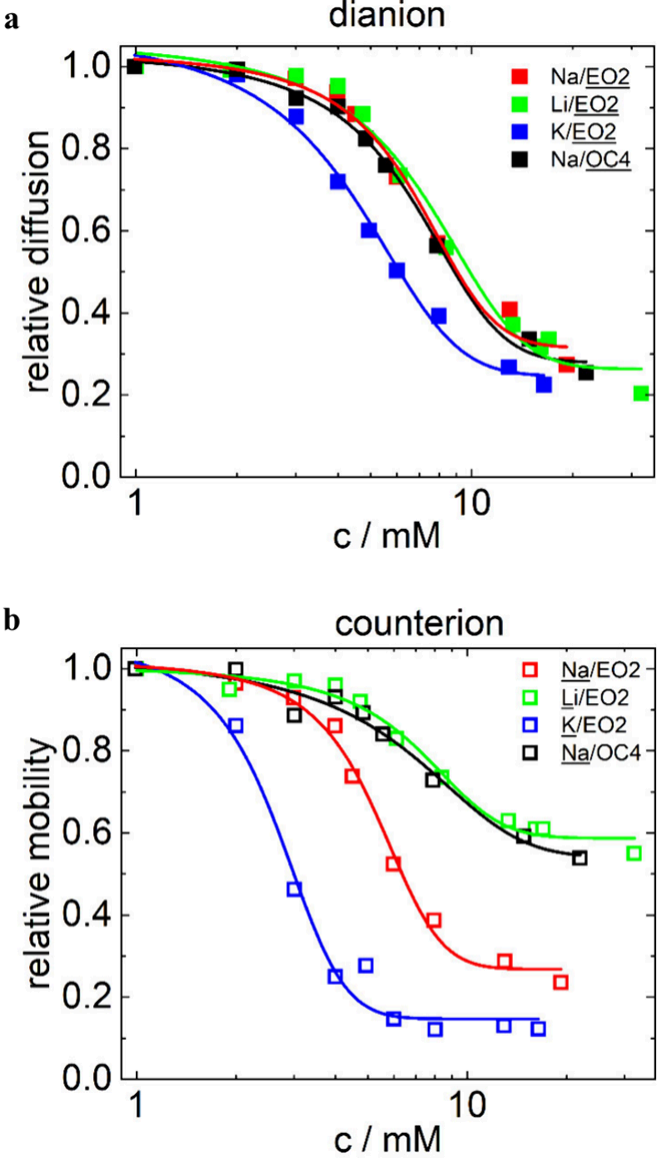
Relative diffusion and mobilities of (a) dianions and (b) corresponding counterions measured at 25 °C of Li2­[B12-EO2-C14] (green), Na2­[B12-EO2-C14] (red), K2­[B12-EO2-C14] (blue), and Na2­[B12-OC4-C14] (black) obtained by ^1^H DOSY NMR for surfactant dianions (filled symbols) and by ^7^Li DOSY NMR for Li^+^ counterions, *T*_1_^23^Na NMR relaxation for Na^+^ counterions, and *T*_2_^39^K NMR relaxation for K^+^ (empty symbols). Further details and comparison are shown in Figure S3. The lines are added to guide the eye. As the relative mobilities of counterions were obtained by diverse NMR techniques, they should be directly compared with caution.

**Figure 3:**
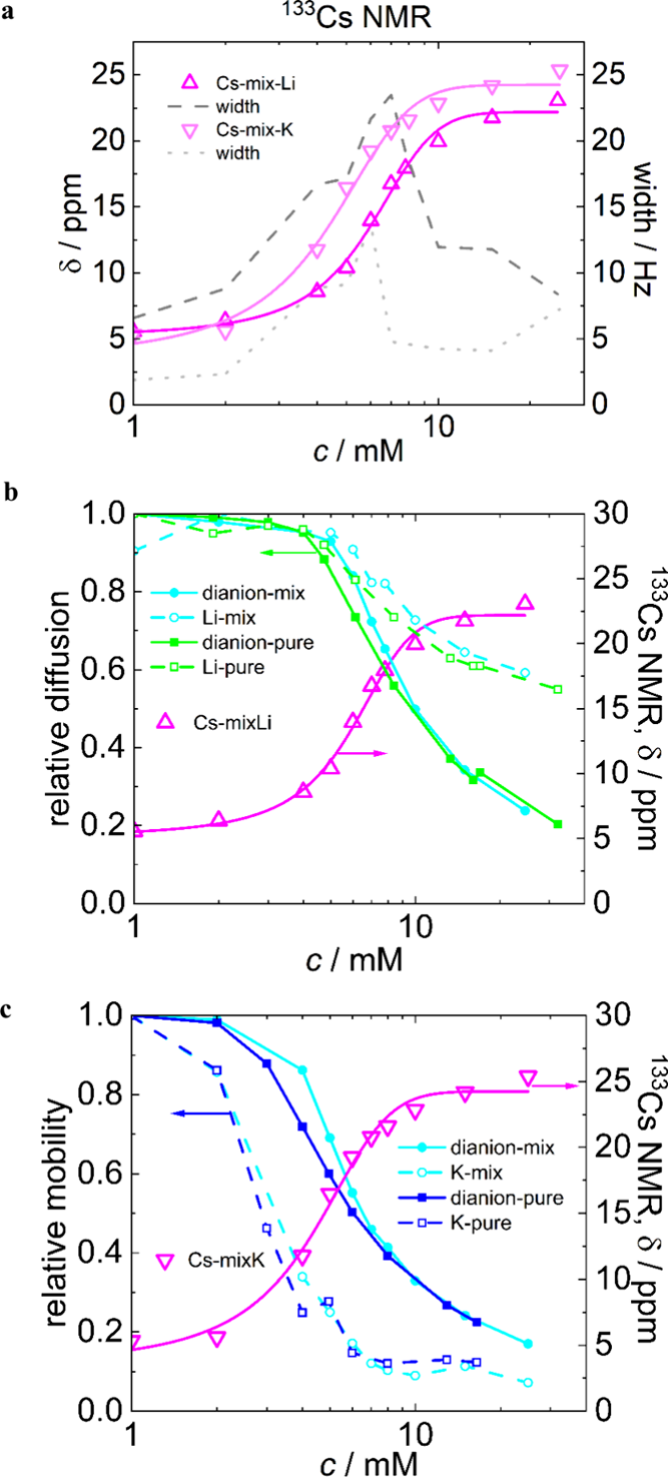
(a) Chemical shifts (δ) and widths of spectral lines measured by ^133^Cs NMR spectroscopy at 25 °C of Li2/Cs­[B12-EO2-C14] (magenta) and K2/Cs­[B12-EO2-C14] (light magenta) cationic mixtures. Concentration dependences of chemical shifts (δ) of ^133^Cs NMR spectra (magenta) and relative diffusion and mobilities determined by ^1^H DOSY (blue, green, and cyan), ^7^Li DOSY (green and cyan), and *T*_2_^39^K NMR (blue and cyan) spectroscopy at 25 °C of (b) Li2/Cs­[B12-EO2-C14] and Li2­[B12-EO2-C14] (cyan for mixtures and green for pure samples) and (c) K2/Cs­[B12-EO2-C14] and K2­[B12-EO2-C14] (cyan for mixtures and blue for pure samples). All of the lines are added to guide the eye.

Therefore, we used techniques sensitive to the diffusion and dynamics of nuclei, which should provide direct information about the micellization process, such as ^1^H DOSY NMR for the determination of the absolute diffusion of surfactant dianions (Figures 2a and 3b,c and Figure S3) and ^7^Li and ^133^Cs DOSY NMR for the calculation of the relative diffusion of respective cations (Figure 2b and Figure S3a). To estimate the relative mobilities of Na^+^ and K^+^, we utilized *T*_1_^23^Na NMR and *T*_2_^39^K NMR relaxations (Figure 2b and Figure S3b-e). Since the various DOSY and relaxation techniques differ in sensitivity from the dynamics of the corresponding species, we interpreted the data on a qualitative level only. While DOSY techniques probe the translational dynamics (diffusion) of the tracked particle, *T*_1_ relaxation techniques are also sensitive to rotational diffusion. In simple cases, these two types of diffusion are linearly related as it was suggested for the Na^+^ cations in our previous study^9^ in line with the paper of D’Agostino.^46^ In contrast, the *T*_2_ relaxation process is more complex due to, for example, possible broadening caused by not only changes in segmental dynamics but also chemical exchange.

Figure 2a shows a substantial decrease in surfactant diffusion upon micellization. All of the studied surfactants appear to follow a similar mechanism of micellization with the exception of the K sample (blue curve), where the aggregation properties are shifted and more pronounced with respect to those of the Li and Na samples. It is interesting to compare the relative mobilities of dianionic surfactants and the corresponding counterions (Figure 2 and Figure S3). In all of the cases, we can see that the dynamics of counterions is slowed by micellization, which directly indicates extensive interaction of counterions with micelles.

Seemingly, Li^+^, Na^+^, and K^+^ mobilities were strongly affected by micellization, but in different ways. As the data were obtained by diverse NMR techniques, it could be treacherous to compare them directly (Figure 2b and Figure S3). Therefore, we used the general term “relative mobility” instead of a “diffusion”. The absolute translational diffusions of the nonaggregated surfactant species are very similar to each other as these have the same or similar molecular weight and size. In contrast, the absolute “mobility” of counterions differs due to diverse hydration and different averaging of free and bound states. The case of Li2­[B12-EO2-C14] should be the most straightforward (Figure S3a), since diffusion of the dianion and counterion was directly determined by ^1^H and ^7^Li DOSY NMR, respectively. The case of Na systems is more complicated (Figure S3b,d,e), but the dynamics of Na^+^ in Na2­[B12-EO2-C14] and Na2­[B12-OC4-C14] samples can be directly compared to each other. Then, we can conclude that the Na^+^ immobilization seen by NMR spectroscopy is ∼50% greater for systems with the EO2 linker than for the OC4-based analogues. This could be related to the different complexation abilities of linkers with different numbers of oxygen atoms, where the linker with three O atoms is more efficient in Na^+^ complexation than that with only two O atoms.

The most puzzling is the behavior of the K2­[B12-EO2-C14] sample (Figure S3c). Again, it seems that the mechanisms of potassium counterion condensation along with the micellization of this sample are different within the alkaline counterion series. We assume that the calculated mobilities of K^+^ based on the width of ^39^K NMR spectral lines (related to *T*_2_ relaxation) might detect any contacts of counterions with surfactant dianions even in premicellar assemblies, thus exaggerating the slowdown effect.

The last system investigated by NMR spectroscopy consisted of the Li and K surfactants containing ca. 8 mol % admixed Cs^+^ (Figure 3). ^133^Cs DOSY NMR was measured only for the most concentrated samples (around 25 mM), and the relative diffusion of Cs^+^ was estimated to be slowed by a factor of 5 with respect to free diffusion in a 100 mM CsCl solution, which is comparable to the decrease in the diffusion of surfactant dianions (Figure 2a). The samples with higher cesium content were not studied because of the insolubility of the Cs salts of *closo*-dodecaborate-based surfactants in water.

Panels b and c of Figure 3 show that the presence of Cs^+^ affected the mobility of Li^+^, Na^+^, and dianionic surfactants only insignificantly in comparison to the pure samples. The relative diffusion of Cs^+^ in the micellar systems was found to be around 5-fold slower than that of freely moving Cs^+^ (dilute CsCl). In this regard, the step-like changes in chemical shifts seen in ^133^Cs NMR spectra (Figure 3a) can be explained by the binding of Cs^+^ to the surfactant micelles. It is worth noting that the curves of ^133^Cs NMR δ in the mixtures with Li and K systems (Figure 3a) are shifted in line with the trends observed for the surfactant dianions of the Li and K samples (Figure 2a). The widths of ^133^Cs NMR spectra (gray lines in Figure 3a) are relatively narrow at low and high concentrations but broadest in the middle region. This again points to the existence of two states of Cs^+^: unbound at low and bound at high concentrations. The actual chemical shift values are a result of averaging.

We took advantage of the fact that all of the NMR techniques used in this study are to a certain extent sensitive either to changes in the mobility of the species or to the formation of multimolecular aggregates and complexes.^9,31^ Accordingly, it was possible to determine the CMC values of all of the samples and complement the output of tensiometry. The evaluation is shown in section 2.3 of the Supporting Information (Figures S5–S7), and it is based on plotting of the NMR data (diffusion, relative mobility, and chemical shift) as a function of a reciprocal concentration. Then, it was possible to distinguish the regions below and above the CMC, and its value was determined by a simple intersection of linear fits of pre- and post-CMC regions. All of the results are listed in Table 2. It is evident that the NMR spectroscopy data confirmed within the experimental error the values, as well as the trends discussed in the tensiometry section above.

**Table 2:** Values of the Critical Micelle Concentration (CMC, mM) of Li2­[B12-EO2-C14], Na2­[B12-EO2-C14], K2­[B12-EO2-C14], and Na2­[B12-OC4-C14] Samples in Water at 25 °C Determined by Tensiometry (pendant drop technique), DOSY, *T*_1_ and *T*_2_ Relaxation, and ^1^H, ^7^Li, ^23^Na, and ^133^Cs NMR Spectroscopies (evaluations in Figures S2 and S5–S7, respectively)

method	Li2/EO2	Na2/EO2	K2/EO2	Na2/OC4
tensiometry	3.4 ± 0.6	2.4 ± 0.9	2.9 ± 0.4	4.1 ± 1.3
NMR for dianion mobility	4.1 ± 1.0	4.0 ± 1.0	2.7 ± 0.3	3.8 ± 1.0
NMR for cation mobility	4.3 ± 0.5	3.5 ± 0.5	1.8 ± 0.5	4.4 ± 0.5
^7^Li NMR	4.2 ± 1.0	–	–	–
^23^Na NMR	–	4.2 ± 0.5	–	4.9 ± 1.0
^133^Cs NMR	3.7 ± 0.5	–	2.7 ± 0.5	–
^1^H NMR (tail)	4.0 ± 0.5	4.0 ± 0.5	2.7 ± 0.3	3.9 ± 0.5
^1^H NMR (head)	3.9 ± 0.5	3.8 ± 0.5	2.9 ± 0.3	4.1 ± 0.5

### MD Simulations of B12-EO2-C14 and B12-OC4-C14 Micelles in Water

To put all of the experimental data mentioned above into context, we performed all-atom MD simulations in explicit water solvents of Li2-, Na2-, K2-, and Cs2­[B12-EO2-C14] and Li2-, Na2-, and K2­[B12-OC4-C14] micelles at a concentration of 100 mM. Typical snapshots are shown in Figures 4 and 5. The results of simulations were further evaluated. Histograms of aggregation numbers (Figures S8 and S9) and corresponding radial distribution functions (RDFs) (Figures S10 and S11) with running integration numbers, *n*(*r*) (Figures 6 and 7), of head–head (B–B), head–counterion (B–X), and linker–counterion (O–X) correlations were calculated for all of the simulated systems. For experimental visualization, characterization, and thermodynamic description of analogical micellar systems, we refer readers to our previous work.^9^

**Figure 4:**
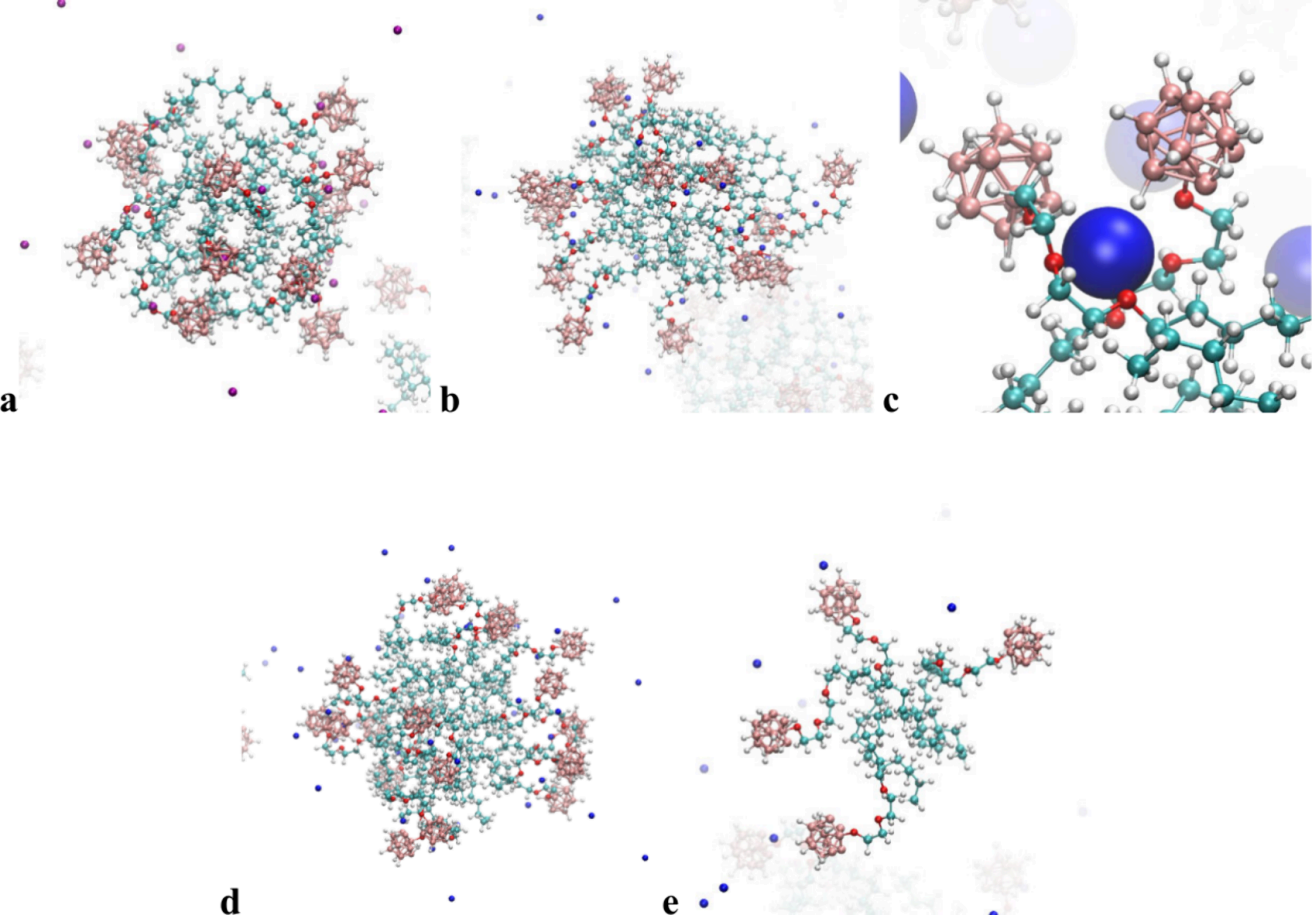
Typical MD simulation snapshots of (a, b, d and e) X2­[B12-EO2-C14] micelles and (c) premicellar aggregates in 100 mM aqueous solutions with counterions (X) as follows: (a) lithium, (b) sodium, (c and d) potassium, and (e) cesium at a concentration 100 mM. Color coding: salmon for B, red for O, cyan for C, white for H, and blue or violet for X.

**Figure 5:**
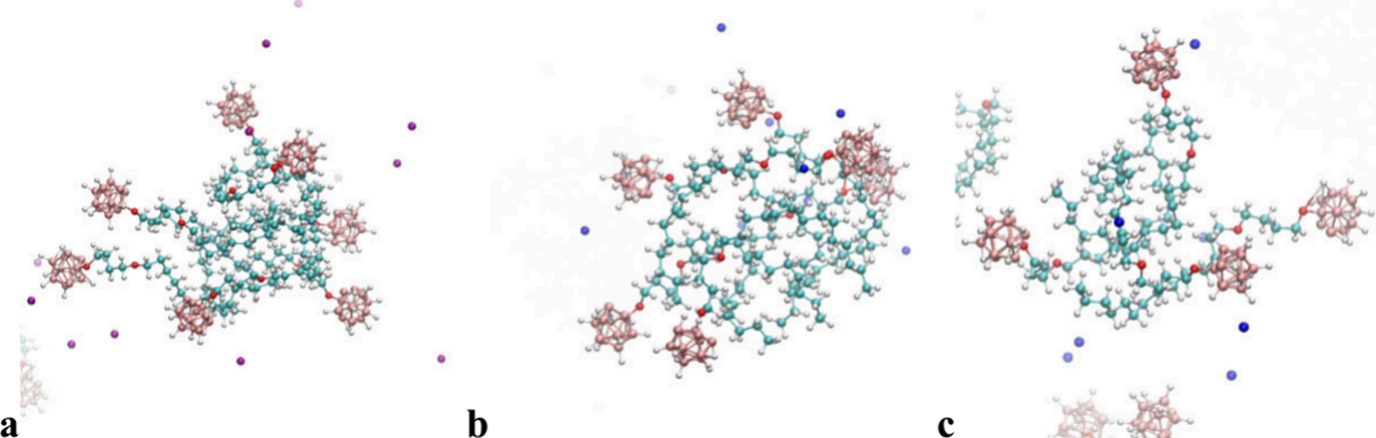
Typical MD simulation snapshots of X2­[B12-OC4-C14] micelles in 100 mM aqueous solutions with counterions (X) as follows: (a) lithium, (b) sodium, and (c) potassium at a concentration of 100 mM. Color coding: salmon for B, red for O, cyan for C, white for H, and blue or violet for X.

**Figure 6:**
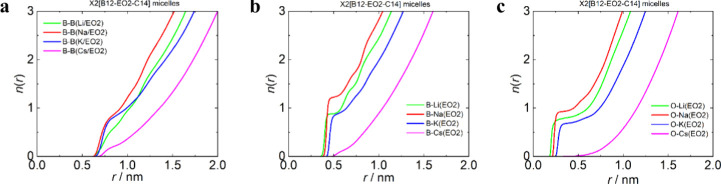
Analysis of the all-atom MD simulation data of Li2­[B12-EO2-C14] (green), Na2­[B12-EO2-C14] (red), K2­[B12-EO2-C14] (blue), and Cs2­[B12-EO2-C14] (magenta) at a concentration of 100 mM. Running integration numbers (*n*(*r*)) of (a) B12 clusters around the central one up to distance *r* of B12···B12 distances (centers of gravity), (b) counterions X (Li, Na, K, and Cs) around the central B12 cluster up to distance *r* of B12···X (centers of gravity), and (c) counterions X (Li, Na, K, and Cs) around the O atom of the linker up to distance *r* of O···X (O closest to the tail).

**Figure 7:**
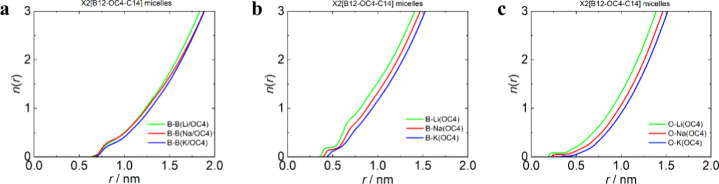
Analysis of the all-atom MD simulation data of Li2­[B12-OC4-C14] (green), Na2­[B12-OC4-C14] (red), and K2­[B12-OC4-C14] (blue) at a concentration of 100 mM. Running integration numbers (*n*(*r*)) of (a) B12 clusters around the central one up to distance *r* of B12···B12 distances (centers of gravity), (b) counterions X (Li, Na, K, and Cs) around the central B12 cluster up to distance *r* of B12···X (centers of gravity), and (c) counterions X (Li, Na, K, and Cs) around the O atom of the linker up to distance *r* of O···X (O closest to the tail).

Evaluation of the data from MD simulations for the series of surfactants with the EO2 linker (Figure 4) indicates the formation of relatively small micelles with aggregation numbers within the range of 5–15, where the Cs micelles are significantly smallest within the series (Figure 4e). Even the morphology of the Cs micelles is different with the linkers and heads stretched toward the bulk solution, while the Li, Na, and K micelles are rather compact with the partly ordered heads located together with collapsed linkers near the hydrophobic core. It is not surprising because the selectivity of the anionic boron cluster “dumbbells” with oligo­(oxyethylene) linkers toward alkaline cations has been described in the literature.^54^

The origin for the morphology of micelles described above can be deduced from RDFs and corresponding running integration numbers (*n*(*r*)) (Figure 6b,c) of counterions around heads and EO2 linkers. While the counterions are strongly correlated with both heads and linkers in Li, Na, and K micelles indicating almost quantitative counterion binding, it is not the case for Cs micelles with mild binding to the heads and almost no contacts with the linkers. In the case of the K system, besides the micelles (Figure 4d), we detected a fraction of surfactant dimers bound together by one K^+^ as shown in Figure 4c.

In general, it seems that the size of the cations is the key factor in the self-assembly of the B12-linker-tail surfactants. While small Li^+^ and Na^+^ cations fit to the loop of EO2 linkers, K^+^ is already relatively large and can be efficiently complexed only by two separate linkers simultaneously. The largest Cs^+^ no longer fits into the EO2 loop. It probably forms contact pairs only with bulky B12 heads and not with linkers because certain contacts of Cs^+^ to the micelles were observed experimentally by ^133^Cs NMR spectroscopy in the cationic blends with Li^+^ or K^+^ (Figure 3).

The micelles of surfactants with OC4 linkers (Figure 5) are in general smaller than their EO2 analogues and exhibit weaker binding of the counterion to both heads and linkers (see the corresponding running integration numbers (*n*(*r*)) in panels b and c of Figure 7). Thus, the morphology is somehow comparable to that of the Cs micelles discussed above (Figure 4e). Despite the correlation of the counterions with the micelles being mild in the OC4 samples, we can see a certain level of selectivity in RDFs even for the OC4-based system. The level of binding to both heads and linkers decreased in the following order: Li^+^ > Na^+^ > K^+^.

To confirm the trends provided by the MD simulations, we determined the size of Li2­[B12-EO2-C14], Na2­[B12-EO2-C14], K2­[B12-EO2-C14], and Na2­[B12-OC4-C14] micelles in the solutions well above the CMC (concentrations around 20–30 mM) by DLS. Since we expected the particles to be in the subnanometer to nanometer range^9^ and the DLS technique is exceptionally sensitive to the presence of much larger dust particles or nanobubbles, which are almost impossible to completely remove even by careful filtering, we performed DLS measurements over the broad range of scattering angles of 50–150 DEG. This provided data of sufficient quality that allowed us to clearly distinguish a fast mode related to the relatively small surfactant micelles. Typical normalized autocorrelation functions are shown in Figure S14, where the mode assigned to the micelles is clearly visible (correlation times of less than 0.1 ms at a scattering angle of 90 DEG) along with slow modes caused by the presence of dust and other intrusions.

Using the Stokes–Einstein equation, we evaluated the hydrodynamic radii (*R*_H_) of the micelles as a function of *q* ^2^, where *q* is the magnitude of the scattering vector (Figure 8), and it is clear that the size of the micelles with radii in the range of 0.7–1.5 nm does not depend on *q* ^2^, which is an indication of the diffusive character of the micelles in line with our previous results.^9^ Although the relatively large experimental error does not allow for strong conclusions, DLS analysis clearly confirmed the presence of micelles with radii around 1 nm, and the obtained data are indicative of the trends discussed above.

**Figure 8:**
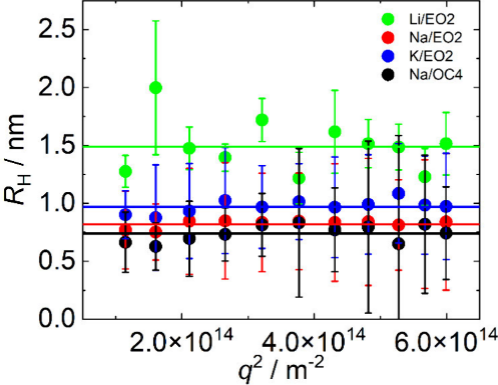
Hydrodynamic radii (*R*_H_) of surfactant micelles vs *q* ^2^ (*q* is the magnitude of the scattering vector) measured at 25 °C of 22 mM Li2­[B12-EO2-C14] (green), 34 mM Na2­[B12-EO2-C14] (red), 27 mM K2­[B12-EO2-C14] (blue), and 31 mM Na2­[B12-OC4-C14] (black), obtained by DLS. The mean values of *R*_H_ are indicated by horizontal lines as follows: 1.49 ± 0.23 nm for Li2­[B12-EO2-C14] (green), 0.82 ± 0.03 nm for Na2­[B12-EO2-C14] (red), 0.97 ± 0.06 nm for K2­[B12-EO2-C14] (blue), and 0.74 ± 0.07 nm for Na2­[B12-OC4-C14] (black).

The impact of counterions on the micellization of the *closo*-dodecaborate-linker-tail surfactants and distinct selectivity to alkaline cations are clearly seen in the histograms of counterions bound to the micelles (Figure 9 and Figures S12 and S13). Because it would be a challenge to unambiguously define a micelle, distinguish which counterions are bound to it, and produce the corresponding histograms, we found an alternative way to analyze the MD simulation data with respect to the number of bound counterions. The histograms were constructed via the criterion based on mutual distances of cations. We assumed that the group of cations close enough to each other (cutoff of 1.3 nm) should share the same micellar aggregate. The drawback of this approach is that we have no information about the aggregation numbers of surfactant dianions in the micelles. Despite this limitation, such histograms are consistent with the results discussed above.

**Figure 9:**
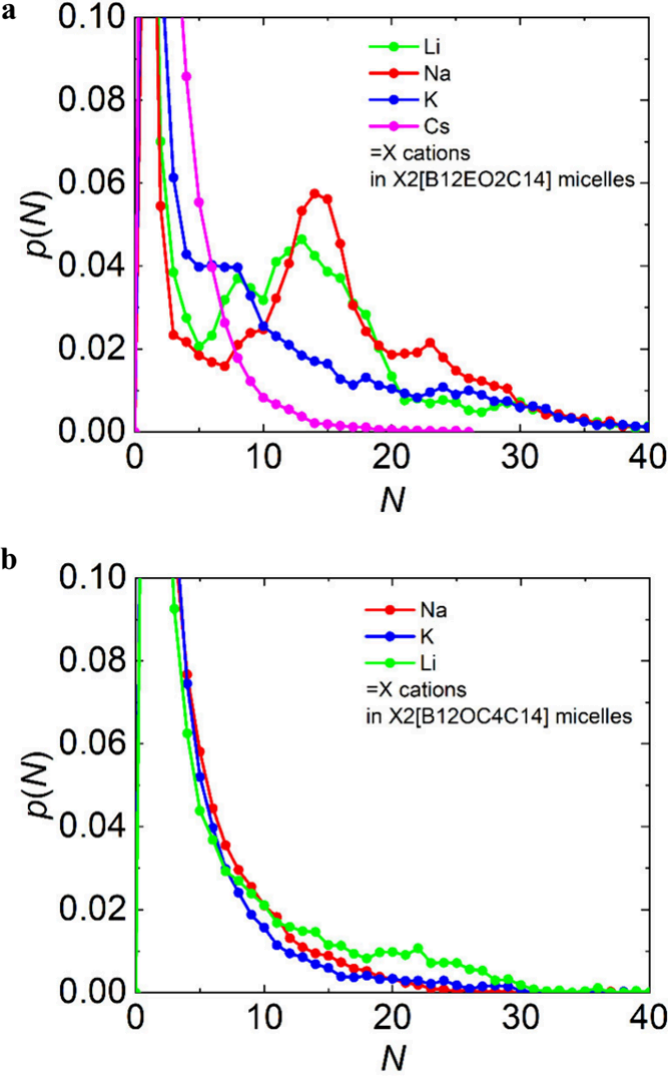
Analysis of the MD simulation data of (a) X2­[B12-EO2-C14] and (b) X2­[B12-OC4-C14] micelles with counterions (X) as follows: lithium (green), sodium (red), potassium (blue), and cesium (magenta) at a concentration 100 mM. Probability *p*(*N*) that the number of counterions in a micelle is equal to *N*, where counterions involved in one micelle were recognized from their distance with a cutoff of 1.3 nm.

For the systems containing EO2 linkers, we clearly see distinct maxima defining the micelles with 10–20 cations only in the case of Li and Na systems (Figure 9a, red and green curves). The graph for K^+^ also shows the maximum, but it is shifted significantly downward (Figure 9a, blue curve), in line with the different character of the K micelles and the abundance of premicellar assemblies (dimers) in this sample. The Cs sample (Figure 9a, magenta curve) as well as all of the OC4-based systems (Figure 9b) did not contain a distinct population of counterions tightly bound to the micelles.

## Conclusions

We designed and synthesized a series of alkaline salts of novel amphiphilic compounds with a bulky dianionic *closo*-dodecaborate head (B12) and a hydrophobic alkoxy tail (C14) connected via short di­(oxyethylene) (EO2) or oxytetramethylene (OC4) linkers. The resulting surfactants exhibited excellent cationic purity without surface-active contaminants. Their self-assembly properties were thoroughly studied by tensiometry, ^1^H, ^11^B, ^7^Li, ^23^Na, ^39^K, and ^133^Cs NMR spectroscopy, and all-atom MD simulations in explicit water.

All of the synthesized samples are surface-active species forming Gibbs monolayers at air–water interfaces. Although the APM and limiting surface tension followed the trends typical for alkaline salts of anionic surfactants (surfactants with the least hydrated counterions occupy the smallest area and are the most surface active, and vice versa), our current study indicates that the capability of linkers to form complexes with the counterions might affect or even reverse this trend. However, further validation with a broader series of alkaline salts and linkers is needed to prove it unambiguously. As a consequence, the surfactant with the OC4 linker was the least surface active and exhibited the highest APM. The formation of compact monolayers was probably tightly related to the binding of the counterions, which efficiently screened highly charged dianionic heads. The EO2 linker (containing three O atoms) was in this regard more efficient for complexation of alkaline cations than the OC4 linker with only two O atoms.

The nature of counterions and their complexation by linkers are probably the key factors for the micellization of the novel surfactants in water. The formation of micelles and the mobility of counterions were monitored by diverse NMR spectroscopy techniques, which provided unique data and valuable insights into the binding of alkaline counterions to the micelles. MD simulations revealed that Li^+^ and Na^+^ cations are extensively bound to the micelles via EO2 linkers, leading to relatively large micelles with a rather compact hydrophilic shell. If the cation-linker contacts did not occur due to the bulkiness of the cation (Cs^+^) or the weak complexation ability of the linker (OC4), the resulting micelles are smaller with the heads and linkers stretched toward the bulk solution. The surfactants with intermediate K^+^ cations exhibited strong binding to EO2 linkers, but a fraction of premicellar aggregates and dimers is assumed. The presence of relatively small micelles in solutions above the CMC of all of the studied samples with a hydrodynamic radius around 1 nm was confirmed by DLS measurements.

As shown previously,^9^ isothermal titration calorimetry is a unique tool for investigation of surfactant (de)­micellization allowing the total thermodynamic description. Therefore, we are planning thorough thermodynamic modeling of the ITC data of the X2­[B12-linker-tail] surfactants to better understand counterion-modulated micellization. The thermodynamic modeling will be complemented by a detailed scattering study to obtain high-quality information about the system.

## Supplementary Material



## Notes

Synthesis, NMR spectroscopy characterization, tensiometry, diffusion and additional spectra measured by NMR spectroscopy, determination of the CMC, size of the micelles measured by DLS, and all-atom MD simulation (PDF)
