# Supplementary material for: Tuning the Surface Activity and Micellization of closo-Dodecaborate-Based Dianionic Surfactants via Linker and Counterion Selection
Source: Langmuir. 2025 Nov 27;41(48):32650–61. doi: 10.1021/acs.langmuir.5c04598 (PMC12874510; doi:10.1021/acs.langmuir.5c04598)
Supplement: Supplementary file 1 [file la5c04598_si_001.pdf]

Supporting Information

Tuning the Surface Activity and Micellization of *Closo*-Dodecaborate-Based Dianionic Surfactants via Linker and Counterion Selection

Belhssen Hleli,[1] Peter Ogrin,[2] Zdeněk Tošner,[3] Žiga Medoš,[2] Tomáš Křížek,[4] Bojan Šarac,[2] Tomaž Urbič,*[2] Marija Bešter-Rogač,*[2] Pavel Matějíček*[1]

[1]*Department of Physical and Macromolecular Chemistry, Faculty of Science, Charles University, Hlavova 2030/8, 128 40 Prague 2, Czech Republic*

[2]*Faculty of Chemistry and Chemical Technology, University of Ljubljana, Večna pot 113, SI-1000 Ljubljana, Slovenia*

[3]*NMR Laboratory, Faculty of Science, Charles University, Hlavova 2030/8, 128 40 Prague 2, Czech Republic*

[4]*Department of Analytical Chemistry, Faculty of Science, Charles University, Hlavova 2030/8, 128 40 Prague 2, Czech Republic*

pavel.matejicek@natur.cuni.cz, marija.bester@fkkt.uni-lj.si, tomaz.urbic@fkkt.uni-lj.si

1 Synthesis and characterization.

1.1 Synthesis and structural analysis of surfactants (NMR analysis).

2 Self-assembly properties.

2.1 Surface activity.

2.2 Diffusion and additional analysis by NMR Spectroscopy.

2.3 Determination of CMC values by NMR spectroscopy.

2.4 Structure of micelles by MD simulations.

2.5 Size of micelles by DLS.

3 References.

1 Synthesis and characterization.

1.1.1. [Bu_4N][$B_{12}H_{11}$-dioxanate] (2)

The synthesis and purification of **2** was carried out and described in our previous work [S1]. Briefly, 1.0 mL of 4 M solution HCl in 1,4-dioxane was added to a round flask under nitrogen along with a suspension of 1.25 g (2.0 mmol) [Bu_4N]$_2$[$B_{12}H_{12}$] (**1a**) and 1.10 g (10.0 mmol) Na[BF_4] in 70 mL of 1,4-dioxane to get 0.8 g as a white product. (Yield 85.1 %)

2: ^{1}H NMR (acetone-d6, ppm): δH 4.52 (4H, m), 3.87 (4H, m), 3.1 (8H, m, [Bu_4N]$^+$), 1.61 (8H, m, [Bu_4N]$^+$), 1.37 (8H, m, [Bu_4N]$^+$), 0.99 (12H, t, [Bu_4N]$^+$); ^{11}B{^{1}H} NMR (acetone-d6, ppm): δB 8.83 (1B), -16.9 (5B), -17.57 (5B), -19.76 (1B); Figure S1a.

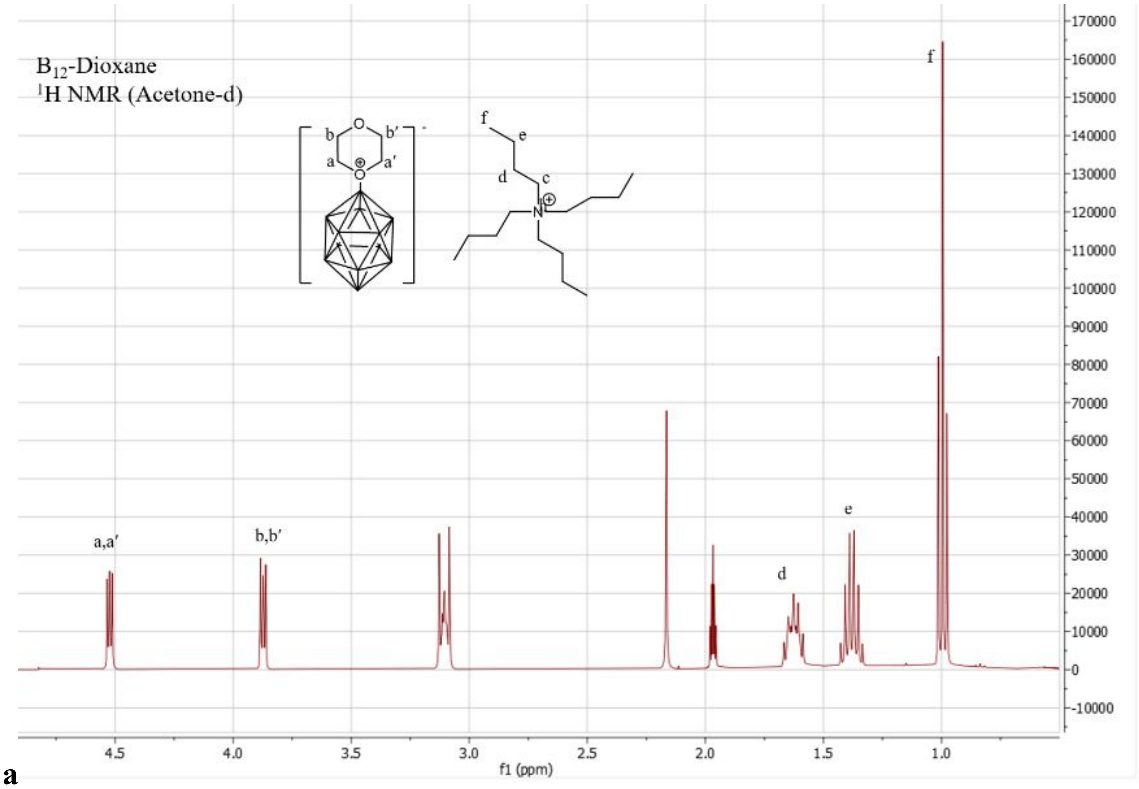

a

Figure S1a. ^{1}H NMR spectrum of [Bu_4N][$B_{12}H_{11}$-dioxanate] (**2**).

1.1.2. [TMA]$_2$[$B_{12}H_{11}$-(OCH_2CH_2)$_2$-O-$C_{14}H_{29}$] (3)

To a solution of 10 mmol of 1-tetradecanol in 25 mL of dry acetonitrile (CH_3CN), 0.4 g of sodium hydride (NaH, 10 mmol) was added carefully under nitrogen. The mixture was stirred at room temperature until hydrogen evolution ceased (~5 min). Then, 1.0 g of compound **2** (2.0 mmol) and 2 mmol of tetrabutylammonium bromide ([TBA]Br) were added to the reaction flask. The mixture was refluxed under nitrogen for 10 h to allow the ring opening via nucleophilic substitution. After cooling to room temperature, the reaction mixture was filtered to remove residual salts, and the solvent was evaporated under reduced pressure to yield a yellowish oil. The crude product was dissolved in a 1:1 MeOH/EtOH mixture, and 2.1 equivalents of

tetramethylammonim chloride ([TMA]Cl) were added. The mixture was stirred until a white precipitate formed, which was collected by filtration. The product was purified by reprecipitation from ethanol (3 times), and dried under vacuum overnight to afford compound **3** as a white solid. (Yield 70 %)

3: ^{1}H NMR (CD_3CN, ppm): δH 3.51 (8H, m), 3.43 (2H, t), 3.12 (3H, s)1.54 (2H, m), 1.3 (22H, m), 0.9 (3H, t); $^{11}B\{^{1}H\}$ NMR (CD_3CN, ppm): δB 6.64(1B), -16.24 (5B), -18.15(5B), -23.37 (1B); Figure S1b.

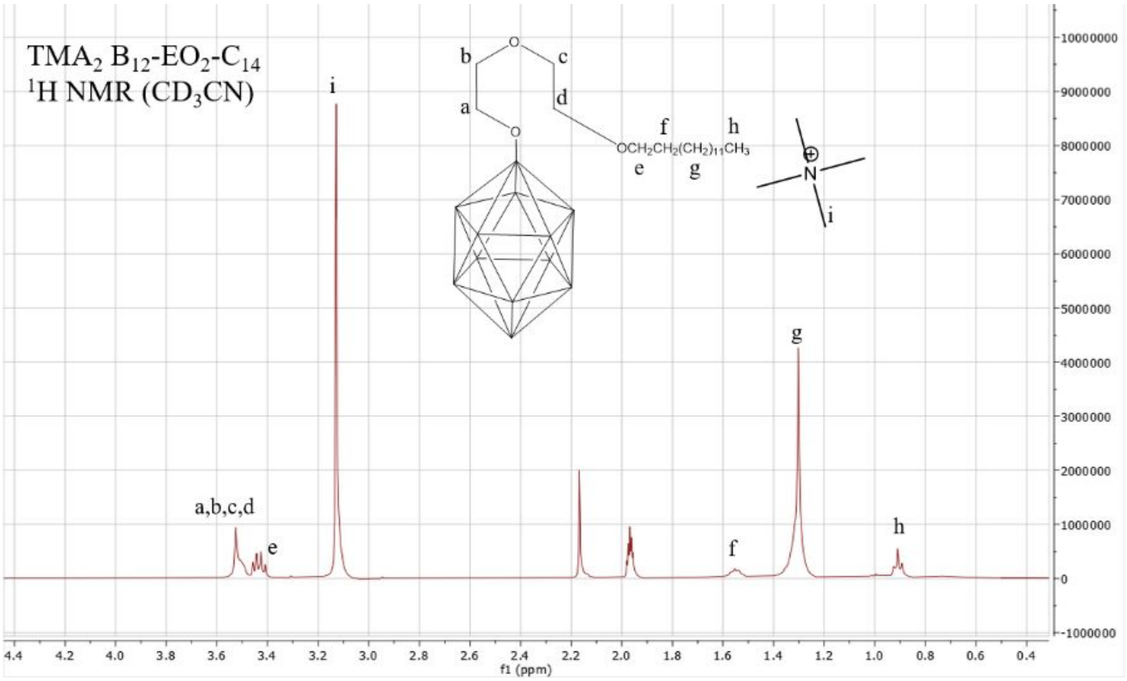

b

Figure S1b. ^{1}H NMR spectrum of $[TMA]_2[B_{12}H_{11}\text{-}(OCH_2CH_2)_2\text{-}O\text{-}C_{14}H_{29}]$ (**3**).

1.1.3. Preparation of $M_2[B_{12}H_{11}\text{-}(OCH_2CH_2)_2\text{-}O\text{-}C_{14}H_{29}]$, M= Na^+, K^+, and Li^+ (4a-c)

The preparation process of $Na_2[B_{12}H_{11}\text{-}(OCH_2CH_2)_2\text{-}O\text{-}C_{14}H_{29}]$ was carried out as follows: the $[TMA]^+$ form was dissolved in CH_3CN:H_2O (1:1) and then passed through a column packed by amberlite Na^+. the eluent was dried under reduced pressure to remove the acetonitrile and the remaining aqueous solution was lyophilized to get **4a** as a white product. (Yield 60 %)

4a: ^{1}H NMR (D_2O, ppm): δH 3.58 (8H, m), 3.46 (2H, t), 1.52 (2H, m), 1.23 (22H, m), 0.81 (3H, t); $^{11}B\{^{1}H\}$ NMR (D_2O, ppm): δB 6.64(1B), -16.24 (5B), -18.34 (5B), -23.37 (1B); Figure S1c.

The preparation process of $M_2[B_{12}H_{11}\text{-}(OCH_2CH_2)_2\text{-}O\text{-}C_{14}H_{29}]$, M= K^+ or Li^+ was done as follows: A column packed by amberlite Na^+ was exchanged to M^+ form (K^+ or Li^+) by passing a MOH solution (KOH or LiOH) through the column, then it was neutralized by adding a HCl solution until pH=7. Once the column is ready, the conversion to the counterion needed was done in the same way as for Na^+ counterion. $K_2[B_{12}H_{11}\text{-}(OCH_2CH_2)_2\text{-}O\text{-}C_{14}H_{29}]$ **4b** white powder (Yield 60 %), and $Li_2[B_{12}H_{11}\text{-}(OCH_2CH_2)_2\text{-}O\text{-}C_{14}H_{29}]$ **4c** white powder (Yield: 60 %).

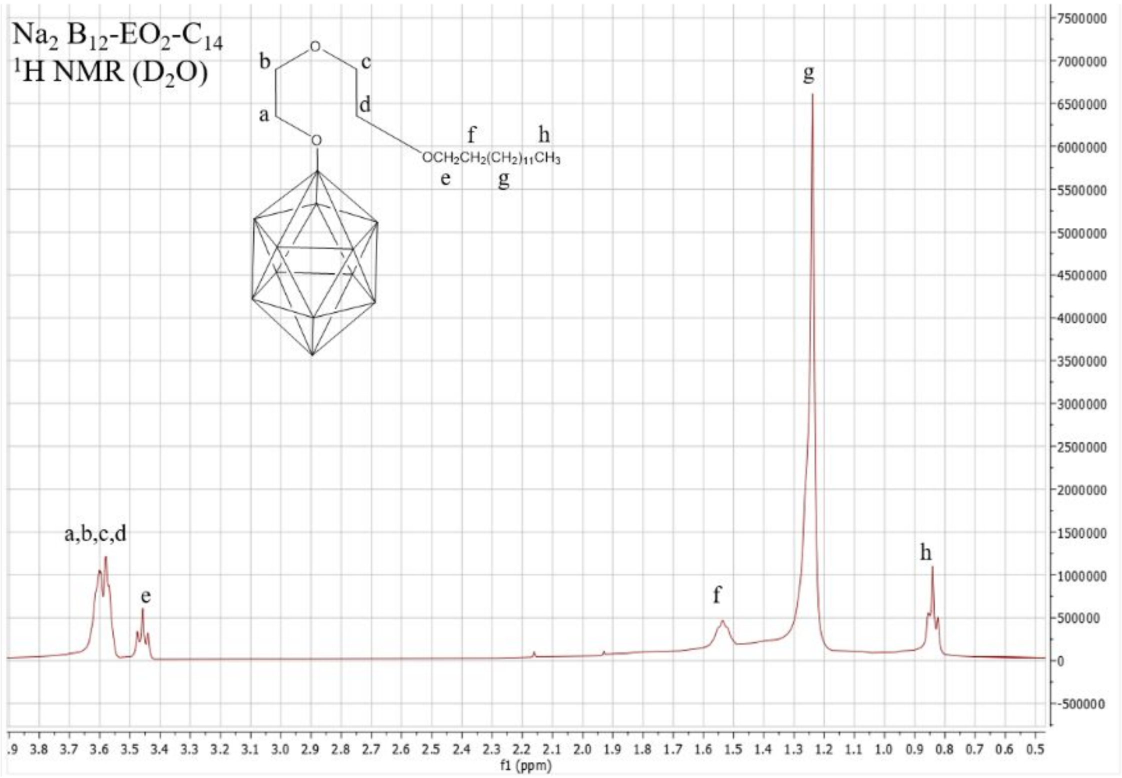

c

Figure S1c. ^{1}H NMR spectrum of $Na_2[B_{12}H_{11}\text{-}(OCH_2CH_2)_2\text{-}O\text{-}C_{14}H_{29}]$ (**4a**).

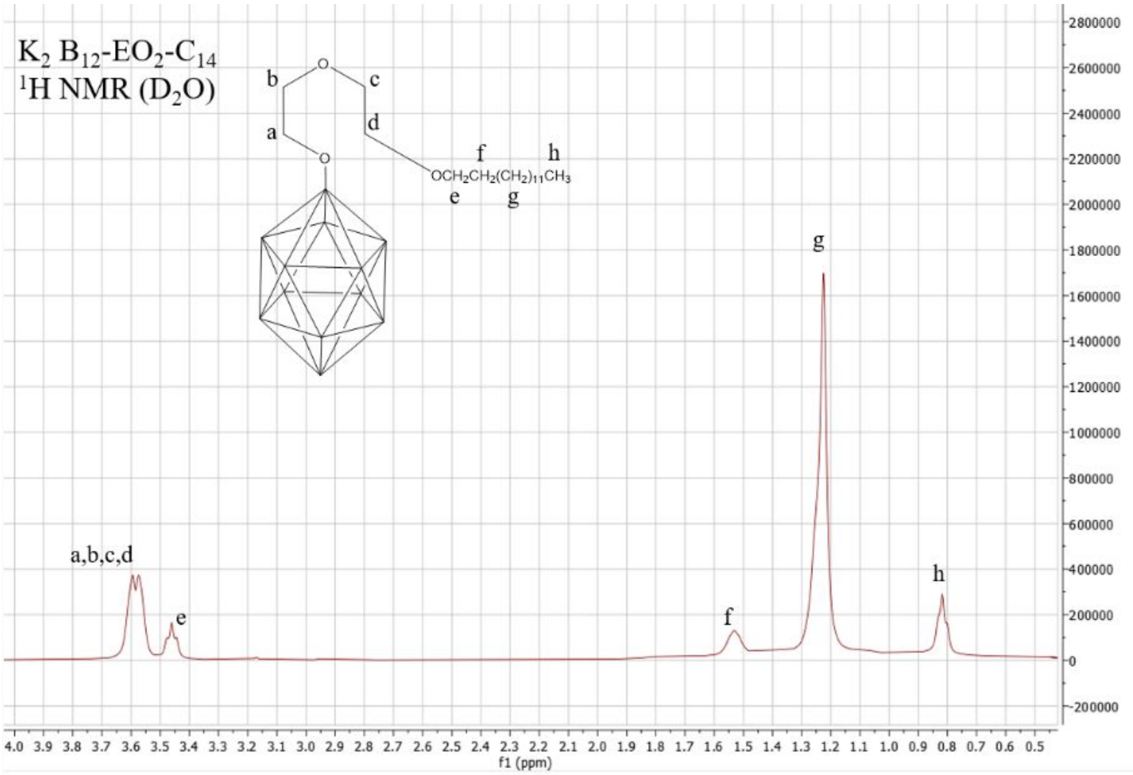

d

Figure S1d. ^{1}H NMR spectrum of $K_2[B_{12}H_{11}\text{-}(OCH_2CH_2)_2\text{-}O\text{-}C_{14}H_{29}]$ (**4b**).

4b: ^{1}H NMR (D_2O, ppm): δH 3.58 (8H, m), 3.45 (2H, t), 1.53 (2H, m), 1.23 (22H, m), 0.83 (3H, t); $^{11}B\{^1H\}$ NMR (D_2O, ppm): δB 6.64 (1B), -16.43 (5B), -18.15(5B), -23.18 (1B); Figure S1d.

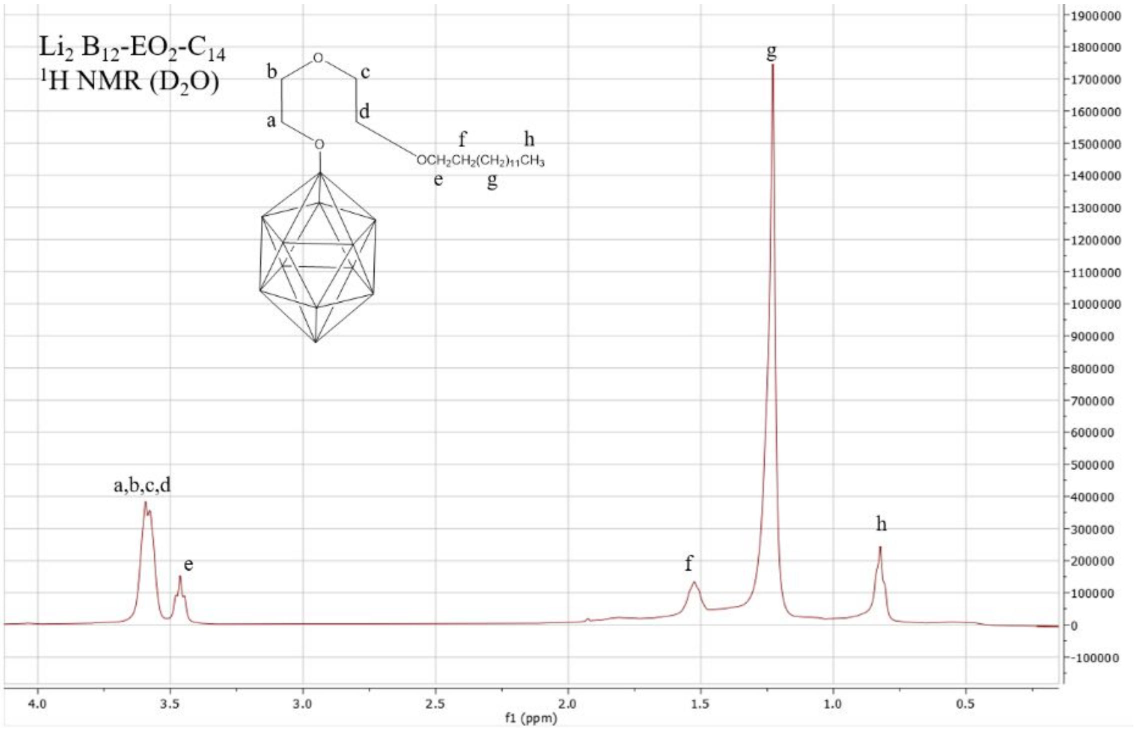

e

Figure S1e. ^{1}H NMR spectrum of $Li_2[B_{12}H_{11}\text{-}(OCH_2CH_2)_2\text{-}O\text{-}C_{14}H_{29}]$ (**4c**).

4c: ^{1}H NMR (D_2O, ppm): δH 3.58 (8H, m), 3.46 (2H, t), 1.52 (2H, m), 1.22 (22H, m), 0.81 (3H, t); $^{11}B\{^1H\}$ NMR (D_2O, ppm): δB 6.64 (1B), -16.24 (5B), -18.34(5B), -23.37 (1B); Figure S1e.

1.1.4. $[Bu_4N][B_{12}H_{11}\text{-THF}]$ (2')

1.48 mL (11.62 mmol) of $BF_3{*}Et_2O$ was added to a solution of 2 g (10.52 mmol) of $Na_2B_{12}H_{12}$ in 50 mL of dry THF in a round flask under nitrogen then stirred at room temperature for 12h. The solution was filtered and dried under reduced pressure. The residue was dissolved in 100 mL of water and treated with a solution of 6.78 g (21.03 mmol) of tetrabutylammonium bromide in 50 mL of water. The precipitate formed was filtered and dried in air to get 3.4 g as a white product. (Yield: 70 %)

2': ^{1}H NMR (acetone-d6, ppm): δH 4.43 (4H, t, $-O(CH_2CH_2)_2)$), 3.11 (8H, t, Bu_4N^+), 2.15 (4H, t, $-O(CH_2CH_2)_2)$), 1.61 (8H, m, Bu_4N^+), 1.38 (8H, m, Bu_4N^+), 0.99 (12H, t, II Bu_4N^+); $^{11}B\{^1H\}$ NMR (acetone-d6, ppm): 6.7 (1B, s), -17.11 (10B, s), -19.94 (1B, s); Figure S1f.

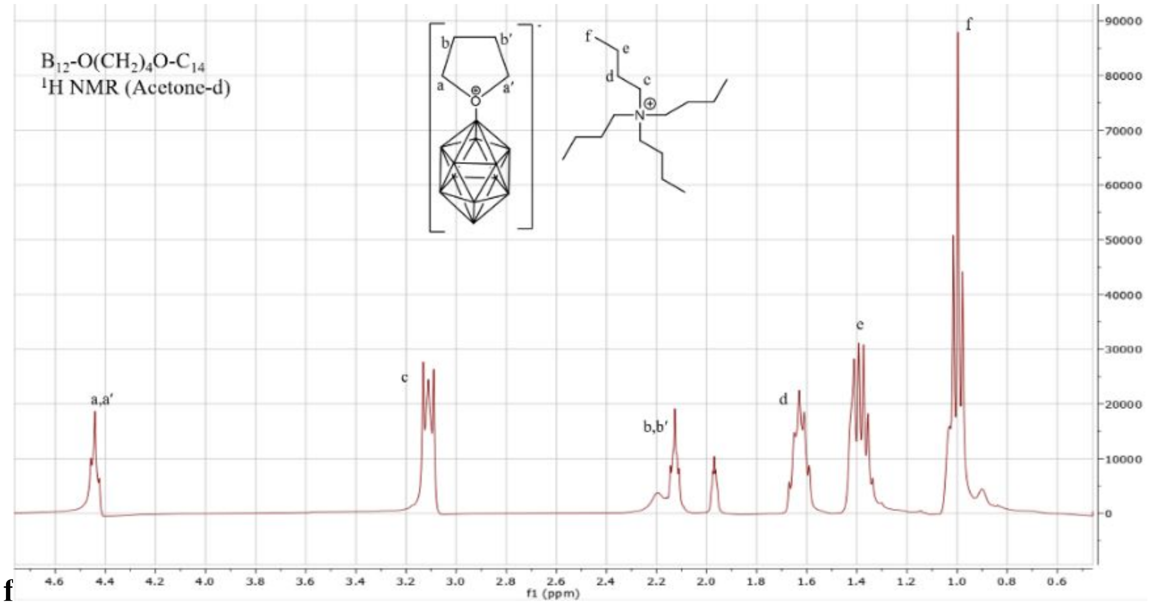

f

Figure S1f. ^{1}H NMR spectrum of [Bu_4N][$B_{12}H_{11}$-THF] (**2'**).

1.1.5. [TMA]$_2$[$B_{12}H_{11}$-O(CH_2)$_4$O-$C_{14}H_{29}$] (3')

To a solution of 11 mmol of 1-tetradecanol in 25 mL of dry acetonitrile (CH_3CN), 0.44 g of sodium hydride (NaH) was added carefully under nitrogen. The mixture was stirred at room temperature until hydrogen evolution ceased (~5 min). Then, 1 g of **2'** (2.2 mol) and 2.2 mol of tetrabutylammonium bromide ([TBA]Br) were added to reaction flask. The mixture was refluxed under nitrogen for 10 hours to allow the ring opening via nucleophilic substitution.

The workup and purification steps were identical to those described for compounds **3** (section 1.1.2), and the product **3'** was obtained as a white solid. (Yield: 72 %)

1.1.6. Preparation of Na_2[$B_{12}H_{11}$-O(CH_2)$_4$O-$C_{14}H_{29}$] (4')

The preparation process of Na_2[$B_{12}H_{11}$-O(CH_2)$_4$-O-$C_{14}H_{29}$] was done following the workup for compound **4**: the [TMA]$^+$ form was dissolved in CH_3CN:H_2O (1:1) and then passed through a column packed by amberlite Na^+. The eluent was dried under reduced pressure to remove the acetonitrile and the remaining aqueous solution was lyophilized to get **4'** as a white product. (Yield: 60 %)

4': ^{1}H NMR (D_2O, ppm): δH 3.43 (6H, m), 1.49 (6H, m), 1.23 (22H, m), 0.83 (3H, t); $^{11}B\{^1H\}$ NMR (D_2O, ppm): δB 6.38 (1B), -16.33 (5B), -18.35(5B), -23.42 (1B); Figure S1g.

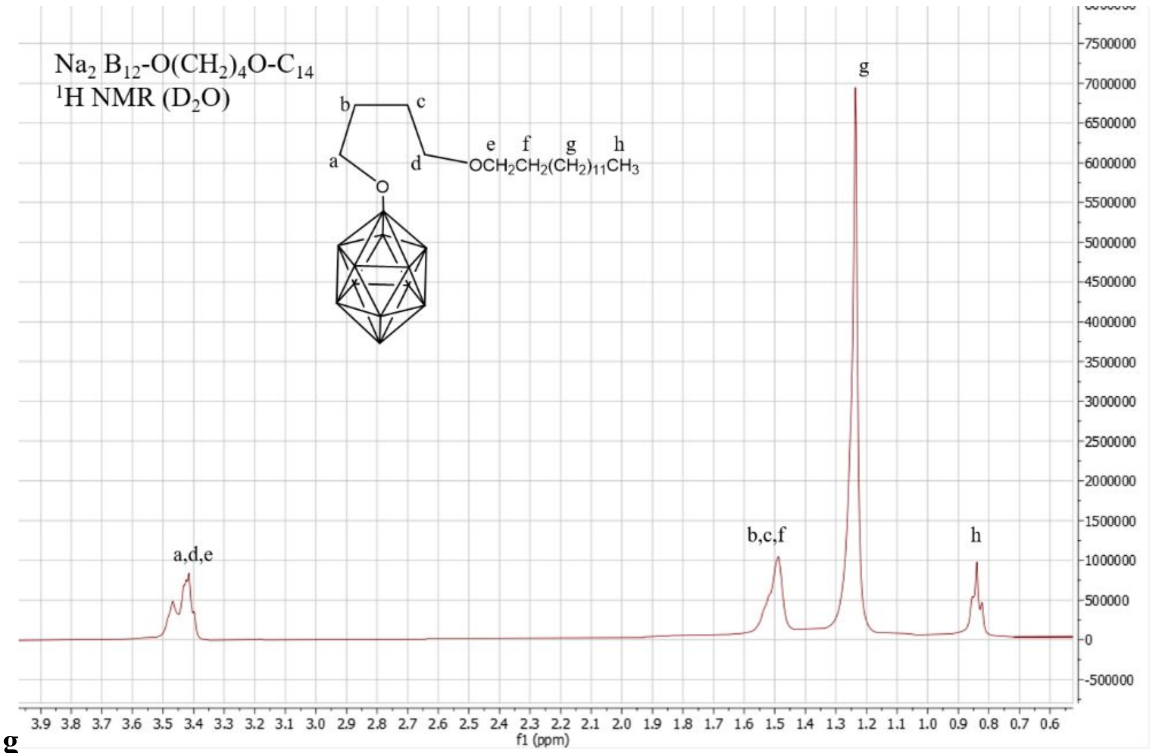

g

Figure S1g. ^{1}H NMR spectrum of $Na_2[B_{12}H_{11}\text{-}O(CH_2)_4O\text{-}C_{14}H_{29}]$ (**4'**).

2 Self-assembly properties.

2.1 Surface activity.

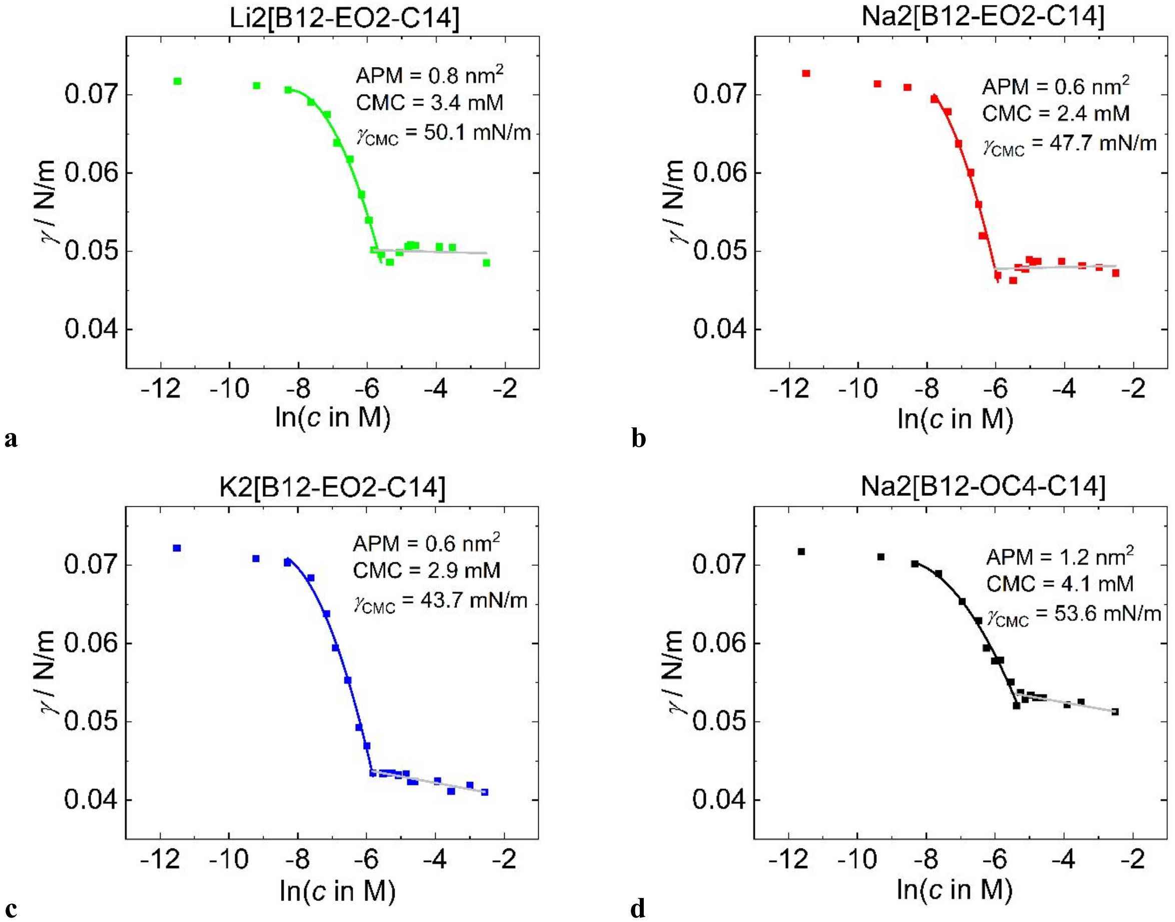

Figure S2. Concentration dependences of surface tension, γ, of (**a**) Li2[B12-EO2-C14], (**b**) Na2[B12-EO2-C14], (**c**) K2[B12-EO2-C14], and (**d**) Na2[B12-OC4-C14] aqueous solutions measured by pendant drop method at 25 °C. Area per molecule, APM, was calculated from the pre-CMC regions and their 2nd order polynomial fits by means of Gibbs adsorption isotherm with n = 3. The values of CMC were estimated by the intersection of lines before and after the break. The values of limiting surface tension γ_{CMC}, was determined as surface tension at CMC.

2.2 Diffusion and additional analysis by NMR Spectroscopy.

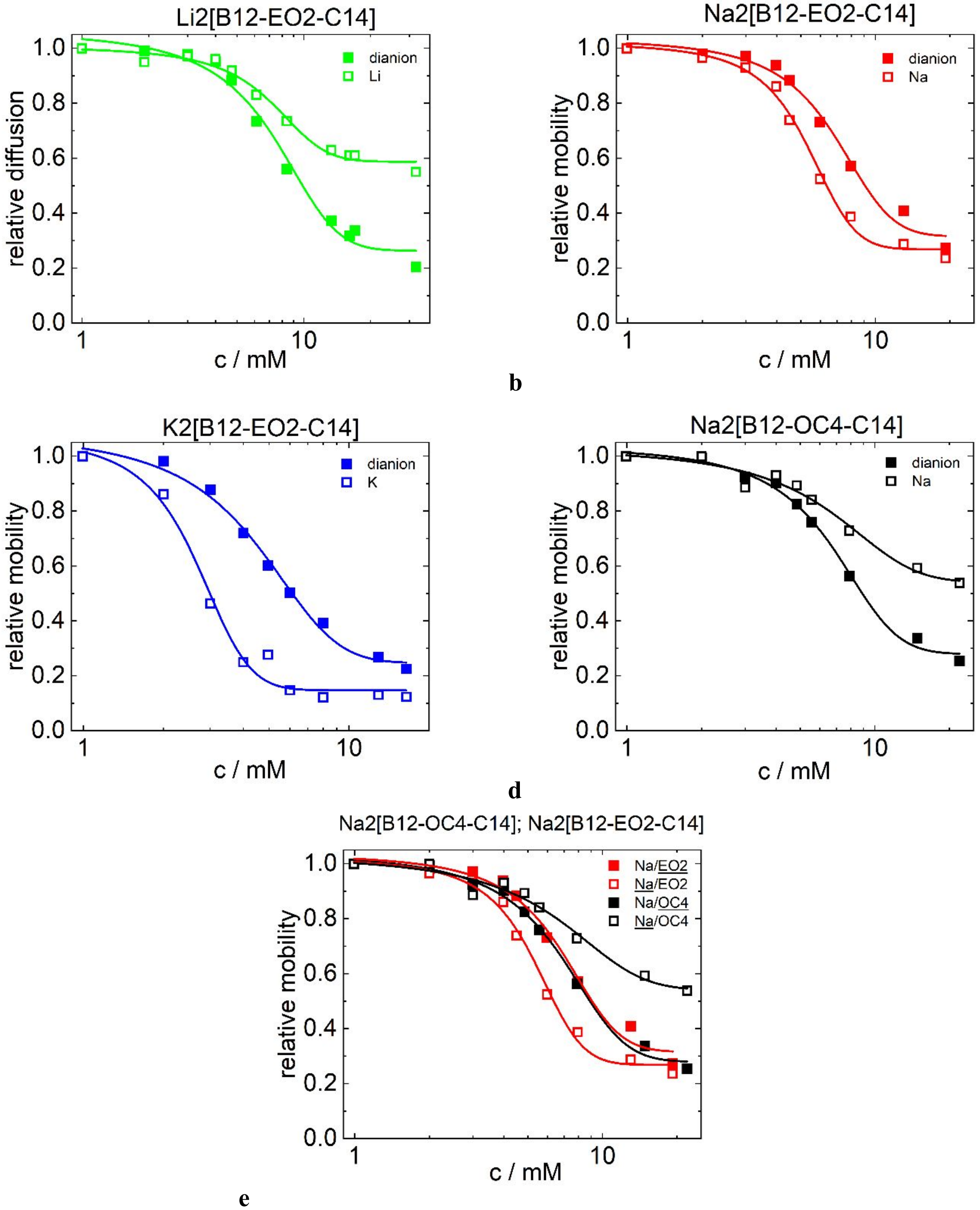

Figure S3. Diffusion coefficients, *D*, measured at 25 °C in aqueous solutions of (**a**) Li2[B12-EO2-C14] (green), (**b**) Na2[B12-EO2-C14] (red), (**c**) K2[B12-EO2-C14] (blue), (**d**) Na2[B12-OC4-C14] (black), and (**e**) comparison of Na2[B12-EO2-C14] and Na2[B12-OC4-C14] obtained by ^{1}H DOSY NMR for surfactant dianions (full symbols), by 7Li DOSY NMR (Li^{+}, green hollow squares), by T_1 ^{23}Na NMR relaxation (Na^{+}, black and red hollow squares), and T_2 ^{39}K NMR relaxation (K^{+}, blue hollow squares). The lines are added to guide the eye. As the relative mobilities of counterions were obtained by diverse NMR techniques, they should be directly compered only with a precaution.

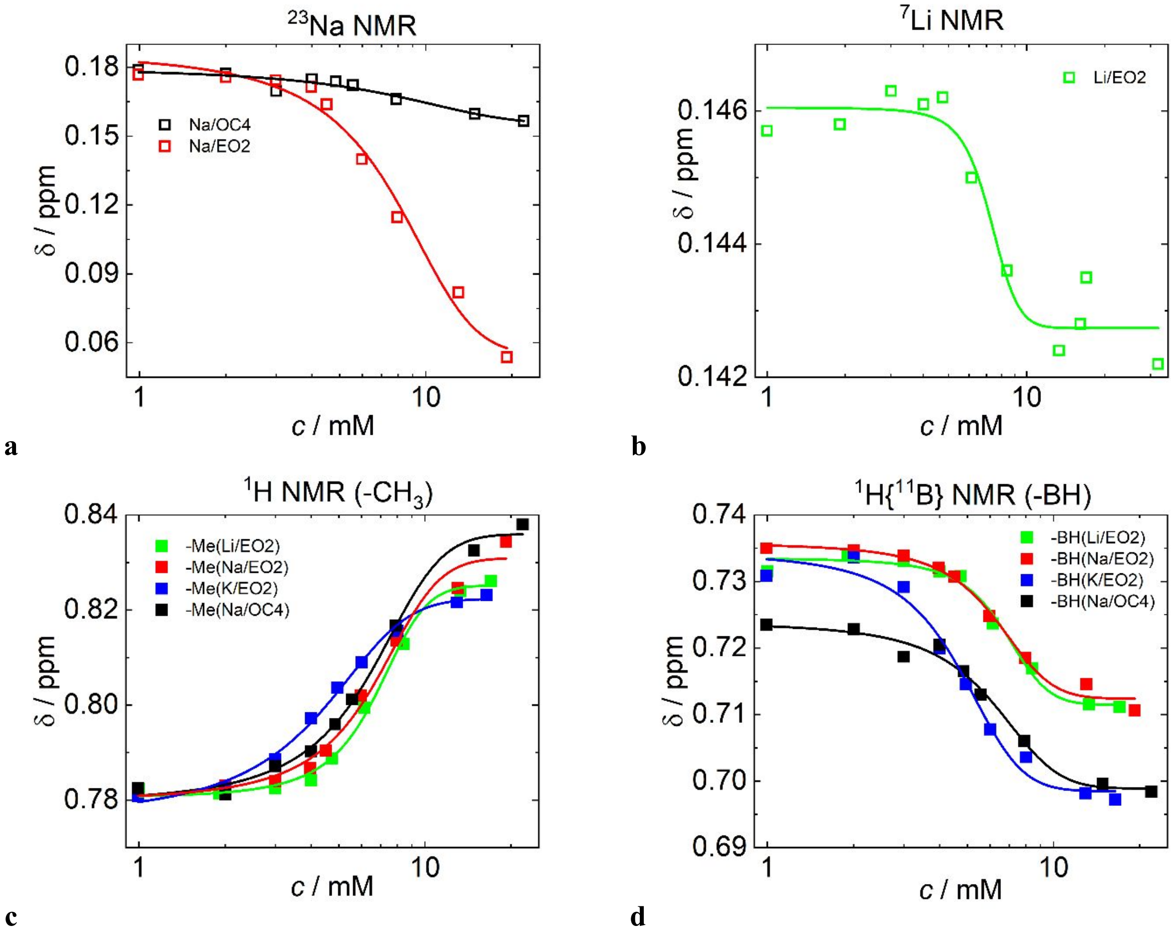

Figure S4. Concentration dependences of chemical shifts, δ, measured at 25 °C in aqueous solutions in (**a**) ^{23}Na NMR spectra of Na2[B12-EO2-C14] (red) and Na2[B12-OC4-C14] (black); (**b**) ^{7}Li NMR spectra of Li2[B12-EO2-C14] (green); (**c**) ^{1}H NMR spectra of $-CH_3$ end groups of Li2[B12-EO2-C14] (green), Na2[B12-EO2-C14] (red), K2[B12-EO2-C14] (blue), and Na2[B12-OC4-C14] (black); and (**d**) ^{1}H{^{11}B} NMR spectra of top B-H vertex of Li2[B12-EO2-C14] (green), Na2[B12-EO2-C14] (red), K2[B12-EO2-C14] (blue), and Na2[B12-OC4-C14] (black). The lines are added to guide the eye.

2.3 Determination of CMC values by NMR spectroscopy.

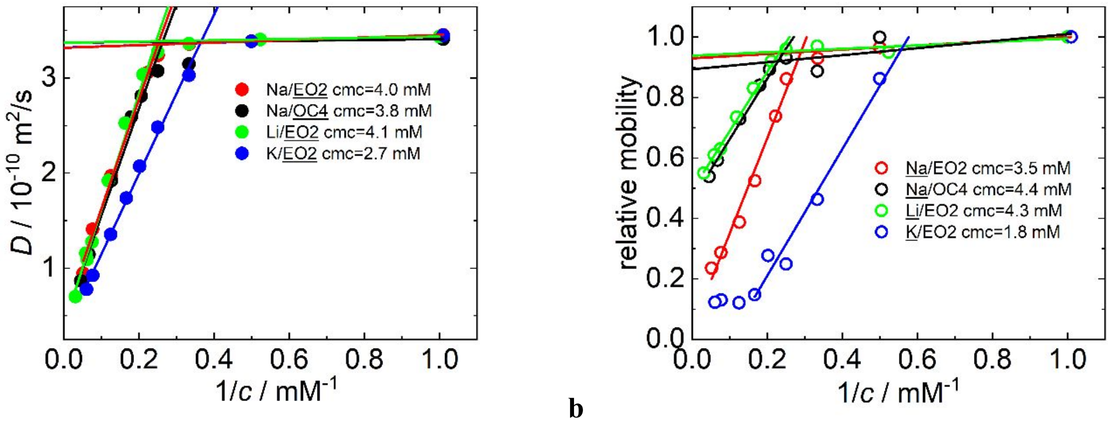

a **b**

Figure S5. Determination of the CMC values from relative diffusion and mobilities *vs.* reciprocal concentration of (**a**) dianions and (**b**) corresponding counterions measured at 25 °C of Li2[B12-EO2-C14] (green squares), Na2[B12-EO2-C14] (red squares), K2[B12-EO2-C14] (blue squares), and Na2[B12-OC4-C14] (black squares) obtained by ^{1}H DOSY NMR for surfactant dianions (full symbols), and by ^{7}Li DOSY NMR for Li^{+} counterions, T_1 ^{23}Na NMR relaxation for Na^{+} counterions and T_2 ^{39}K NMR relaxation for K^{+} (hollow symbols). The CMC values are determined as an intersection of linear fits before and after the CMC.

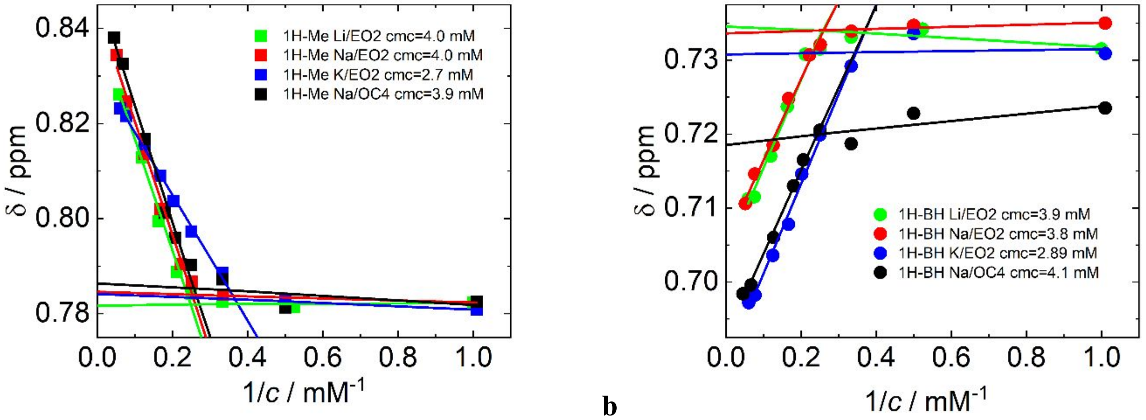

a **b**

Figure S6. Determination of the CMC values from dependences of chemical shifts, δ, *vs.* reciprocal concentration measured at 25 °C in aqueous solutions from (**a**) ^{1}H NMR spectra of $-CH_3$ end groups of Li2[B12-EO2-C14] (green), Na2[B12-EO2-C14] (red), K2[B12-EO2-C14] (blue), and Na2[B12-OC4-C14] (black); and (**b**) ^{1}H{^{11}B} NMR spectra of top B-H vertex of Li2[B12-EO2-C14] (green), Na2[B12-EO2-C14] (red), K2[B12-EO2-C14] (blue), and Na2[B12-OC4-C14] (black). The CMC values are determined as an intersection of linear fits before and after the CMC.

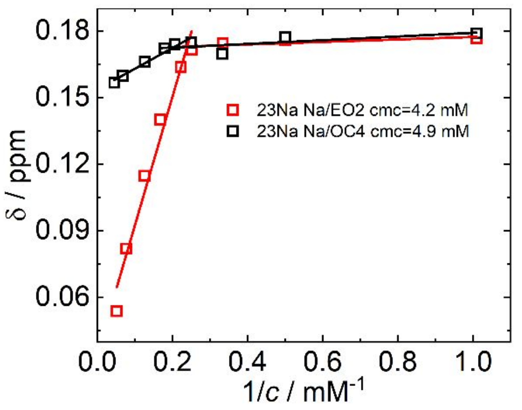

a

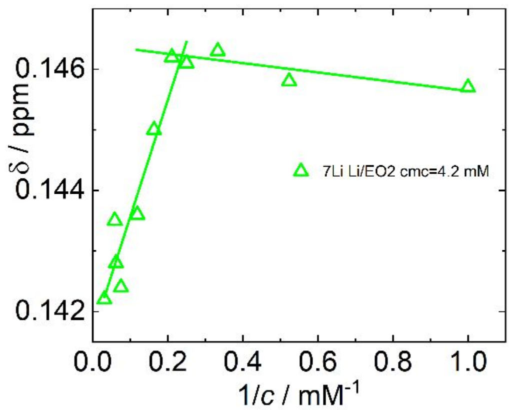

b

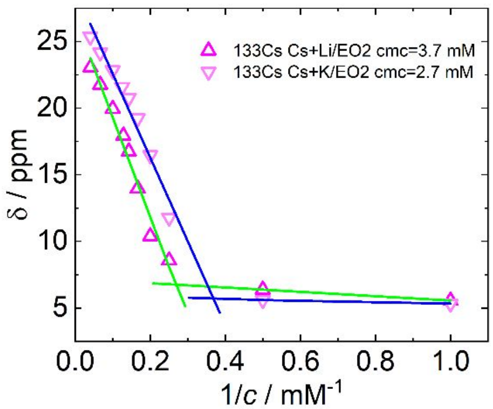

c

Figure S7. Determination of the CMC values from dependences of chemical shifts, δ, *vs.* reciprocal concentration measured at 25 °C in aqueous solutions from (**a**) ^{23}Na NMR spectra of Na2[B12-EO2-C14] (red) and Na2[B12-OC4-C14] (black); (**b**) ^{7}Li NMR spectra of Li2[B12-EO2-C14] (green); (**c**) ^{133}Cs NMR spectra of Li2/Cs[B12-EO2-C14] (magenta triangles) and K2/Cs[B12-EO2-C14] (light magenta triangles) mixtures with Cs-content estimated by CZE to be 8 mol-% for both samples. The CMC values are determined as an intersection of linear fits before and after the CMC.

2.4 Structure of micelles by MD simulations.

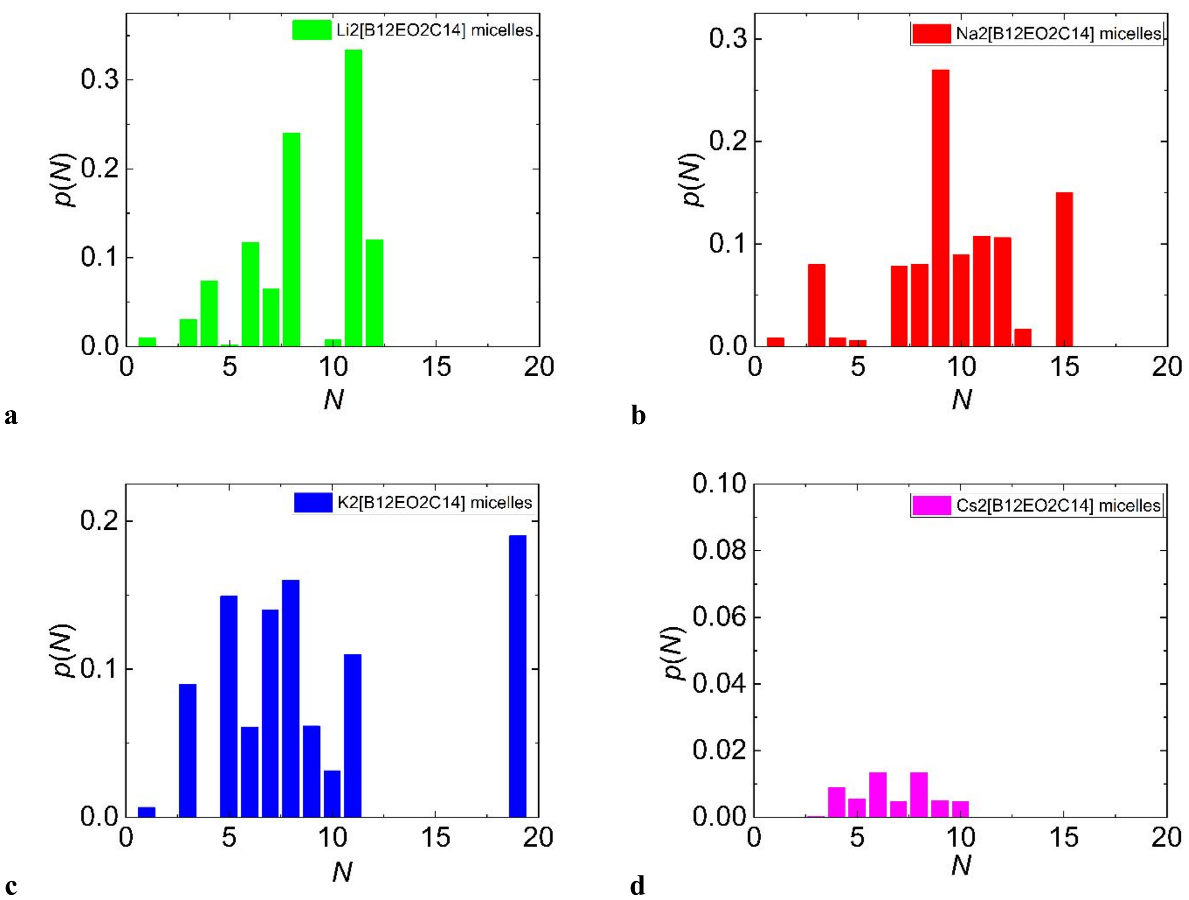

Figure S8. Analysis of the all-atom MD simulation data of (**a**) Li2[B12-EO2-C14] (green), (**b**) Na2[B12-EO2-C14] (red), (**c**) K2[B12-EO2-C14] (blue), and (**d**) Cs2[B12-EO2-C14] (magenta), *c*=100 mM: the histogram of probability *p*(*N*) that the surfactants dianion is in micelle with aggregation number *N*, where the surfactant molecules involved in the aggregation was recognized from the tail-to-tail distance with cut-off 0.5 nm.

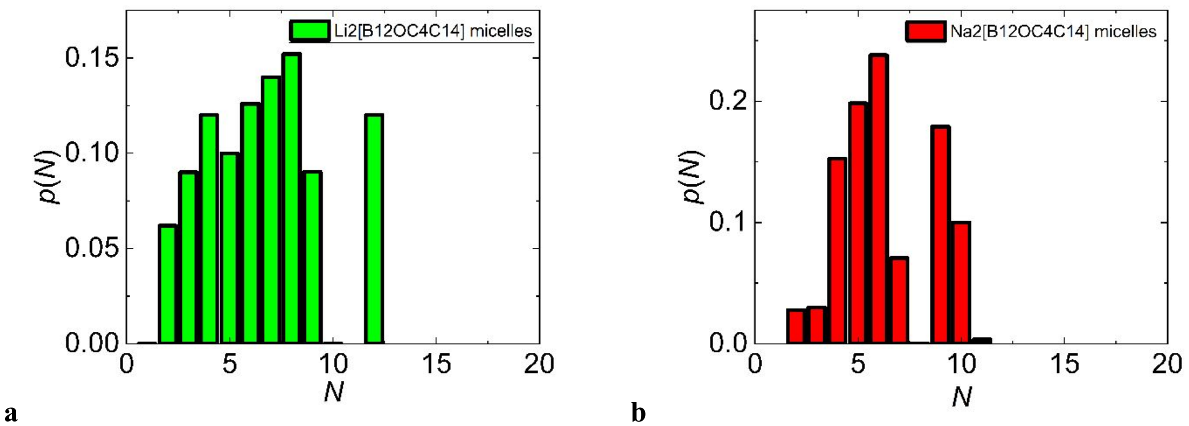

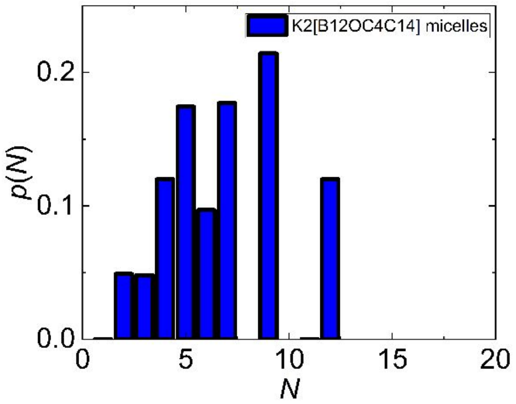

c

Figure S9. Analysis of the all-atom MD simulation data of (**a**) Li2[B12-OC4-C14] (green), (**b**) Na2[B12-OC4-C14] (red), and (**c**) K2[B12-OC4-C14] (blue), *c*=100 mM: the histogram of probability *p*(*N*) that the surfactants dianion is in micelle with aggregation number *N*, where the surfactant molecules involved in the aggregation was recognized from the tail-to-tail distance with cut-off 0.5 nm.

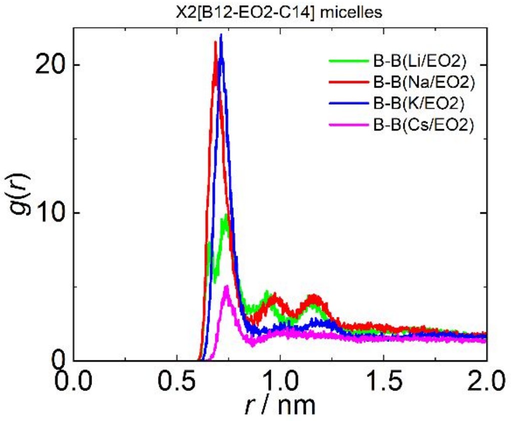

a

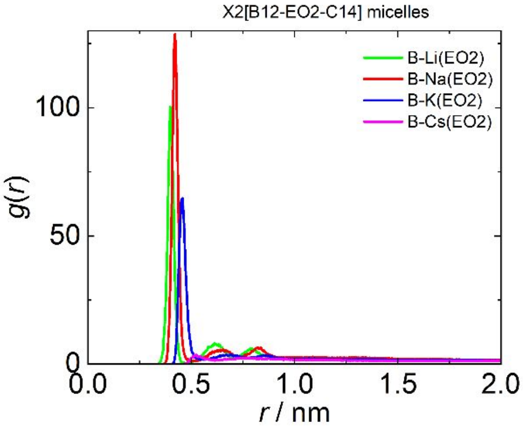

b

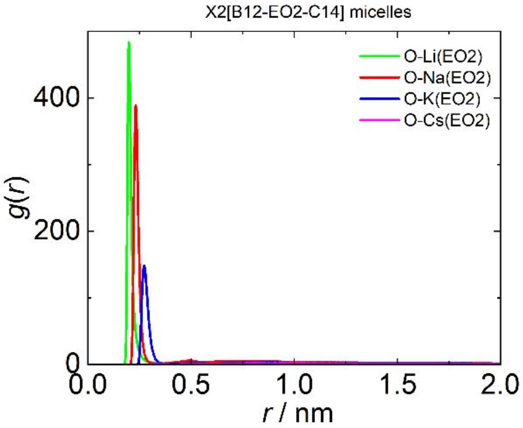

c

Figure S10. Analysis of the all-atom MD simulation data of Li2[B12-EO2-C14] (green), Na2[B12-EO2-C14] (red), K2[B12-EO2-C14] (blue), and Cs2[B12-EO2-C14] (magenta), *c*=100 mM: radial distribution function, *g*(*r*), of (**a**) B12 clusters around the central one up to distance *r* of B12...B12 distances (centers of gravity), of (**b**) counterions X (Li, Na, K, Cs) around the central B12 cluster up to distance *r* of B12...X (centers of gravity), and of (**c**) counterions X (Li, Na, K, Cs) around O atom of the linker up to distance *r* of O...X (O closest to tail).

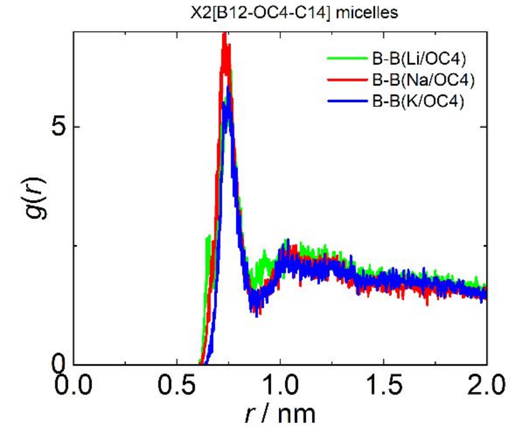

a

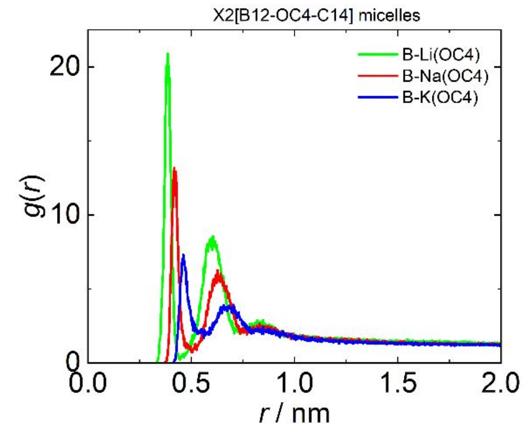

b

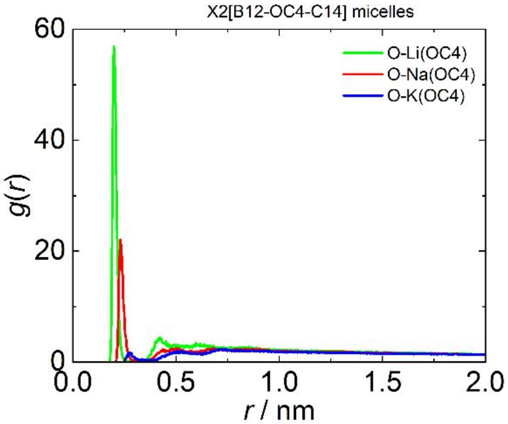

c

Figure S11. Analysis of the all-atom MD simulation data of Li2[B12-OC4-C14] (green), Na2[B12-OC4-C14] (red), and K2[B12-OC4-C14] (blue), *c*=100 mM: radial distribution function, *g*(*r*), of (**a**) B12 clusters around the central one up to distance *r* of B12...B12 distances (centers of gravity), of (**b**) counterions X (Li, Na, K, Cs) around the central B12 cluster up to distance *r* of B12...X (centers of gravity), and of (**c**) counterions X (Li, Na, K, Cs) around O atom of the linker up to distance *r* of O...X (O closest to tail).

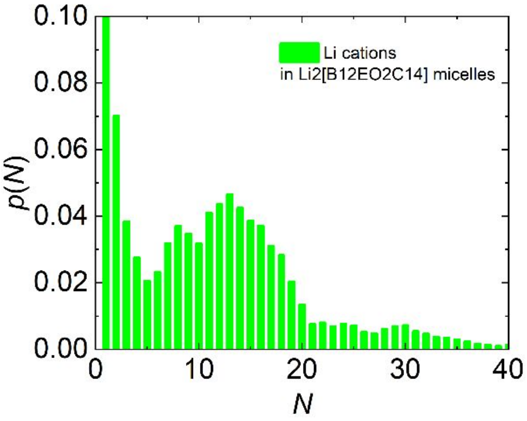

a

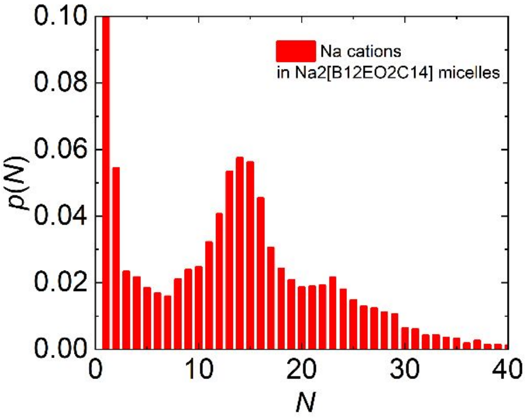

b

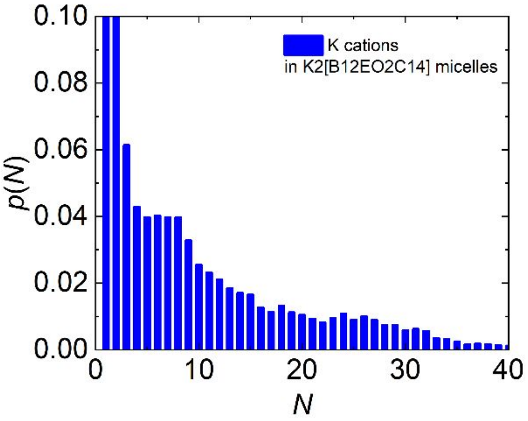

c

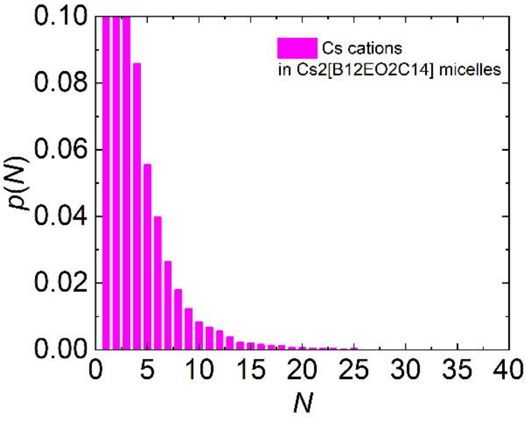

d

Figure S12. Analysis of the all-atom MD simulation data of (**a**) Li2[B12-EO2-C14] (green), (**b**) Na2[B12-EO2-C14] (red), (**c**) K2[B12-EO2-C14] (blue), and (**d**) Cs2[B12-EO2-C14] (magenta), c=100 mM: the histogram of probability $p(N)$ that the number of counterions in a micelle is equal to N, where counterions involved in one micelle was recognized from their distance with cut-off 1.3 nm.

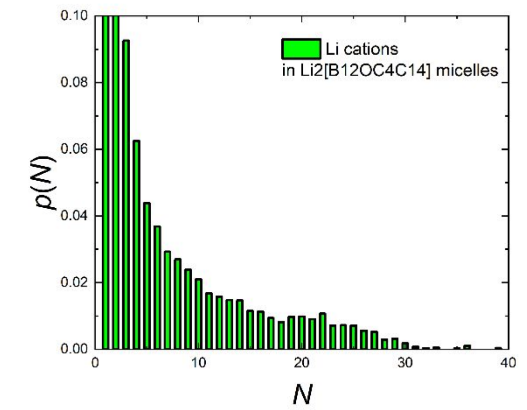

a

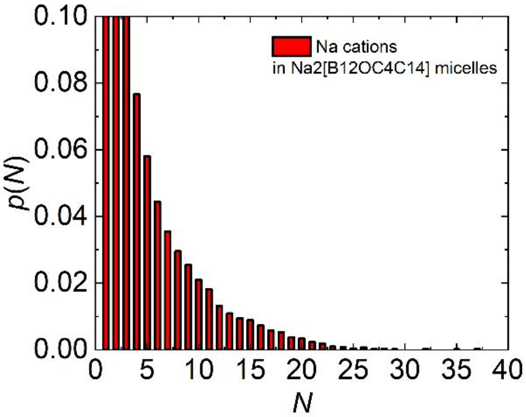

b

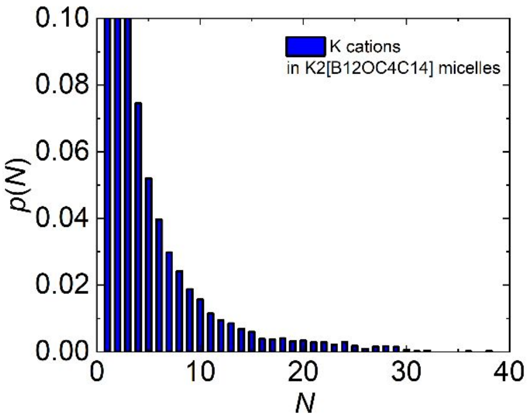

c

Figure S13. Analysis of the all-atom MD simulation data of (**a**) Li2[B12-OC4-C14] (green), (**b**) Na2[B12-OC4-C14] (red), and (**c**) K2[B12-OC4-C14] (blue) c=100 mM: the histogram of probability $p(N)$ that the number of counterions in a micelle is equal to N, where counterions involved in one micelle was recognized from their distance with cut-off 1.3 nm.

2.5 Size of micelles by DLS.

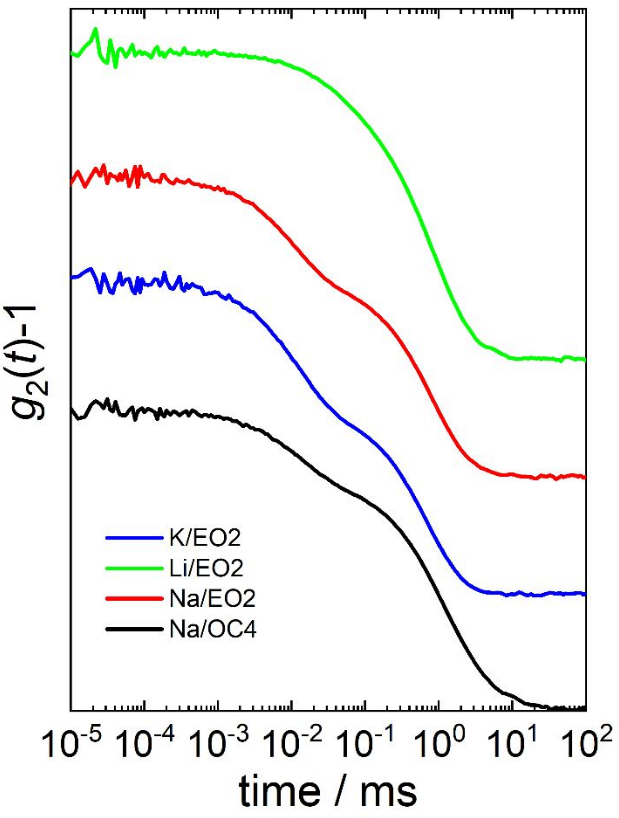

Figure S14. Typical examples of normalized autocorrelation functions, $g_2(t)$, of the surfactant micelles measured by DLS at scattering angle 90DEG at temperature 25 °C of Li2[B12-EO2-C14] (green), Na2[B12-EO2-C14] (red), K2[B12-EO2-C14] (blue), and Na2[B12-OC4-C14] (black).

3 References.

[S1] Hleli, B.; Medos, Z.; Ogrin, P.; Tosner, Z.; Kereiche, S.; Gradzielski, M.; Urbic, T.; Bester-Rogac, M.; Matejicek, P. Closo-dodecaborate-based dianionic surfactants with distorted classical morphology: Synthesis and atypical micellization in water. *J. Coll. Interface Sci.* **2023**, *648*, 809-819, https://doi.org/10.1016/j.jcis.2023.06.013.
